# Harnessing the potential of plant transcription factors in developing climate resilient crops to improve global food security: Current and future perspectives

**DOI:** 10.1016/j.sjbs.2021.01.028

**Published:** 2021-01-20

**Authors:** Rahil Shahzad, Shakra Jamil, Shakeel Ahmad, Amina Nisar, Zarmaha Amina, Shazmina Saleem, Muhammad Zaffar Iqbal, Rana Muhammad Atif, Xiukang Wang

**Affiliations:** a Agricultural Biotechnology Research Institute, Ayub Agricultural Research Institute, Faisalabad 38000, Pakistan; b State Key Laboratory of Rice Biology, China National Rice Research Institute, Hangzhou 310006, China; c Department of Plant Breeding and Genetics, University of Agriculture, Faisalabad 38000, Pakistan; d Center for Advanced Studies in Agriculture and Food Security (CAS-AFS), University of Agriculture Faisalabad, University Road, 38040, Faisalabad, Pakistan; e College of Life Sciences, Yan’an University, Yan’an 716000, China

**Keywords:** Biotic stress, Abiotic stress, Climate change, Plant transcription factors, Food security, Crop improvement

## Abstract

Crop plants should be resilient to climatic factors in order to feed ever-increasing populations. Plants have developed stress-responsive mechanisms by changing their metabolic pathways and switching the stress-responsive genes. The discovery of plant transcriptional factors (TFs), as key regulators of different biotic and abiotic stresses, has opened up new horizons for plant scientists. TFs perceive the signal and switch certain stress-responsive genes on and off by binding to different *cis*-regulatory elements. More than 50 families of plant TFs have been reported in nature. Among them, DREB, bZIP, MYB, NAC, Zinc-finger, HSF, Dof, WRKY, and NF-Y are important with respect to biotic and abiotic stresses, but the potential of many TFs in the improvement of crops is untapped. In this review, we summarize the role of different stress-responsive TFs with respect to biotic and abiotic stresses. Further, challenges and future opportunities linked with TFs for developing climate-resilient crops are also elaborated.

## 1 Introduction

Agricultural crops are important as they represent the largest source of calories (70–80%) and protein (60–70%) intake for mankind. However, the changing climate is adversely affecting plant health and causing food insecurity due to outbreak of multiple biotic and abiotic stresses (Mall et al., 2017). Plants have adopted different resistance mechanisms for survival under changing environmental conditions. For example, in response to drought stress, plants start developing a strong root system and promote lateral roots to increase the water catchment area. Similarly, in response to terminal heat stress, plants shift their growth patterns from vegetative to reproductive growth to limit the effect of terminal heat stress on reproduction; many other similar examples exist (Lan Thi Hoang et al., 2017). Plants respond to different biotic and abiotic challenges by modulation of molecular, cellular, biochemical, and physiological responses. In many cases, the driving forces behind these changes are genes encoding transcription activators and repressors that regulate expression of downstream stress responsive genes and modulate different developmental and metabolic pathways (Tolosa and Zhang, 2020). During the past couple of decades, extensive research has focused on the identification of the key factors associated with regulating the molecular response to stress signal perception (Lan Thi Hoang et al., 2017).

**Fig. 1:**
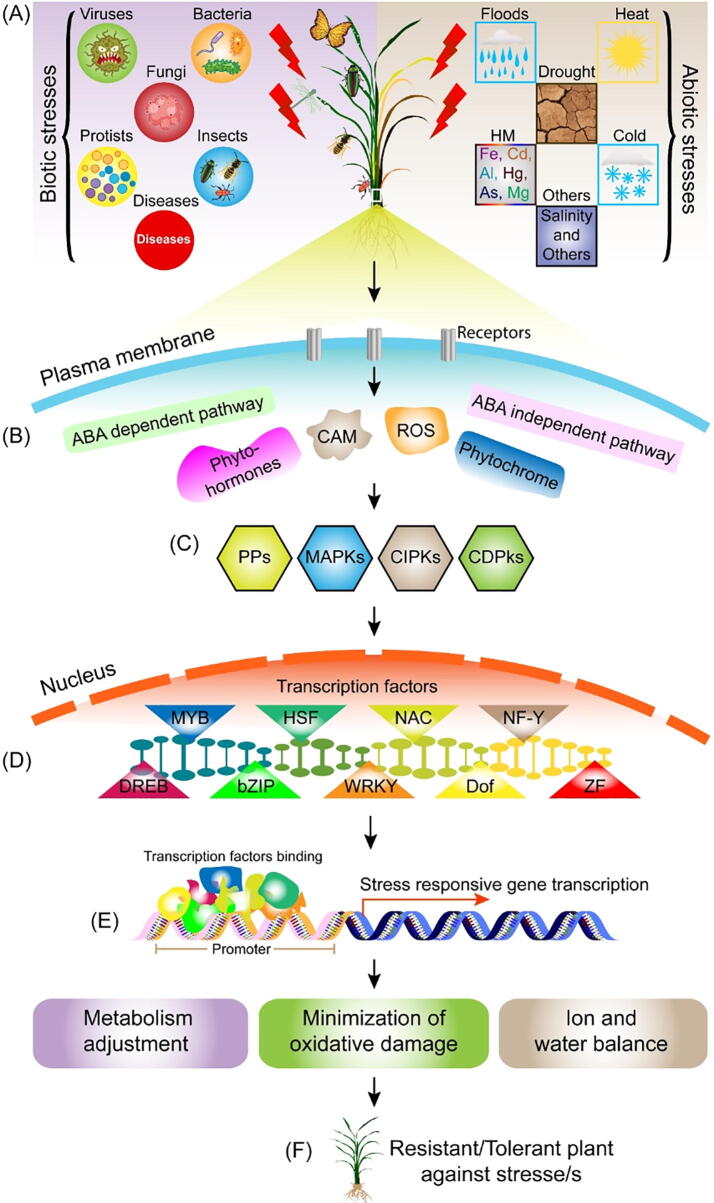
Mechanism of action of transcriptional factors (TFs) for development of resistance in plants against biotic and abiotic stresses. (**A**) Different biotic and abiotic stresses affect plant growth and development; however, plants have developed rapid response strategies to unfavorable conditions; these involve interconnected networks at the molecular level controlled by signal cascades. The different components of stress responses are (**B**) signal perception, and (**C**) signal transduction, (**D**) transcriptional regulation, (**E**) gene expression, (**F**) gene adoption. When plant cells perceive a stress signal, receptors or sensors in the cell wall or membrane detect the stress stimulus, followed by a rapid response that transduces the external signal to intracellular signals. Signal cascades involving intracellular molecules or ions are activated along with kinase cascades, which are generally cytoplasmic. Major cascades are associated with reactive oxygen species (ROS) and calcium ions (Ca^2+^). Phytohormones, including abscisic acid, jasmonic acid, salicylic acid, and ethylene, are powerful second messengers that coordinate signal transduction pathways during stress responses. These signals activate several parallel transduction pathways, which often involve phosphatases and protein kinases. Following the initial step of signal perception, plants activate two major signal cascades: the mitogen-activated protein kinase (MAPK) and calcium-dependent protein kinase (CDPK) pathways. Finally, specific TFs are upregulated or downregulated by protein kinases or phosphatases, and the TFs bind to *cis*-elements of stress-responsive genes to enhance or suppress their transcription. Finally, stress resistant/tolerant plants emerge.

Transcriptional factors (TFs) are frontline defensive factors of plants against various biotic and abiotic stresses (Fig. 1). These play fundamental roles in plant tolerance/resistance to various biotic and abiotic stresses (Lan Thi Hoang et al., 2017, Javed et al., 2020). TFs usually respond to stress by binding their target sites within *cis*-acting elements in promoter regions of stress responsive genes (Fig. 1E). TFs binding in promotor regions initiate a complex formation for biochemical, physiological, and molecular responses. The stress response comprises of signal perception, signal transduction, and expression of stress-responsive genes (Fig. 1B–D). The stress signal is received by receptors in plant cell membranes, or the cell wall and transduced through intracellular elements, i.e., Ca^2+^, Reactive Oxygen Species (ROS), phytochromes, phosphatases, and protein kinases to TFs. TFs then control gene expression and initiate expression of stress responsive genes (Erpen et al., 2018).

Plant genomes contain a large complement of TF genes; approximately 6% of total expressed sequence tags (ESTs) (Table S1). However, the major roles under biotic and abiotic stresses are played by dehydration responsive element binding (DREB), basic leucine zipper (bZIP) domain, MYB, no apical meristem (NAM), ATAF1/2, and cup-shaped cotyledon (CUC2) (NAC), heat shock factors (HSF), DNA-binding with one ZF-proteins (Dof), WRKY, Nuclear factor Y (NF-Y), and Zinc-fingers. Meanwhile, TFs have become core part of plants’ research due to huge variation in responses elicited and potent role in both biotic and abiotic stress tolerance such as, WRKY TFs simultaneously regulate drought, heat, cold stress, counter disease, as well as pest and nematode attacks (Jiang et al., 2017).

Keeping in view of the above mentioned facts, we have summarized the current status of different plant TF classes, including DREB, bZIP, MYB, NAC, Zinc-finger, HSF, Dof, WRKY, and NF-Y, and their substantial role in biotic and abiotic stress responses which may facilitate development of resistant and/or tolerant crop plants. Moreover, different crop improvement techniques, particularly gene editing technique is proposed as one of the potential tools for crop improvement by editing TFs. Further, current associated challenges and future opportunities are listed as well.

## 2 Structure, function, and mechanism of action of various groups of TFs

**Fig. 2:**
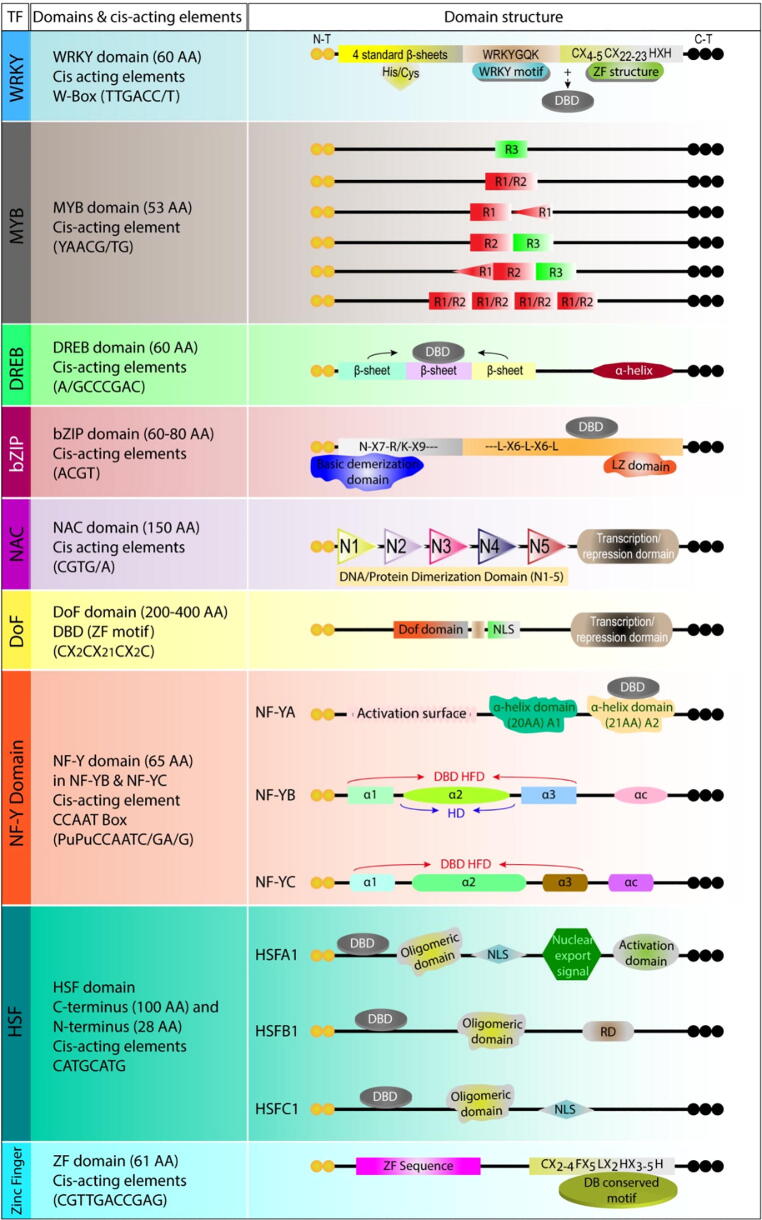
Illustration of domains’ structure, composition, and *cis*-regulatory elements of nine TFs including WRKY, MYB, DREB, bZIP, NAC, Dof, NF-Y, HSF, and Zinc finger. **WRKY:** The WRKY TFs contains the N-terminal WRKYGQK domain, while at the C-terminal, Zinc Finger (ZF) motifs are present. The ZF-motif may be either Cx_4-5_Cx_22-23_HxH or Cx_7_Cx_23_HxC. The WRKY domain spans around 60 amino acids and is a DNA binding protein, which binds to W-BOX (TTGACT/C) and many other binding sites (Eulgem et al., 2000, Ülker and Somssich, 2004, Van Verk et al., 2008, Rushton et al., 2010, Rushton et al., 2012). **MYB:** The MYB domain consists of 52 amino acids repeats forming 3α-helicase, in which the second and third helicase form helix structure with three equally spaced tryptophan, forming hydrophobic core in a three-dimensional (3D) helix structure. The third helix is the “recognition helix” that directly binds to DNA and inserts it into a major grove. Two MYB repeats are bind in the major grove and recognize specific DNA target sequence during DNA contact (Dubos et al., 2010, Zhong and Ye, 2015). **DREB:** The DBD of DREB family members is the AP_2_/ERF type with a conserved region of 60 amino acids; AP_2_ family members have α-helix and β-sheet stretches at a highly conserved region, the later within the DBD. DREB proteins attach with C-repeat sequence (A/GCCGAC) or dehydration responsive elements (DRE) for activation of stress responsive genes (Fujita et al., 2005, Sharoni et al., 2011, Chen et al., 2016). **bZIP:** The bZIP domain is made up of a basic region at the N-terminal linked to C-terminal leucine zipper. About 16 amino acids are present in the basic region, which form an invariant motif (N-x_7_-R/K) that is responsible for binding to DNA. The bZIP domain consists of two structures: N-x_7_-R/K-x_9_ (DNA binding site) and leucine zipper (hydrophobic amino acids, i.e., Val, Met with heptad repeats of Leu) (Liao et al., 2008, Banerjee and Roychoudhury, 2017). **NAC:** The NAC domain spans approximately 150 amino acids, and has five conserved sub-domains (N_1_–N_5_) that form motifs for protein–protein interaction, DNA binding, or TF dimerization. Structural studies have shown that DBD is located at N-terminal while regulatory domain is located at the C-terminal (Baillo et al., 2019, Yuan et al., 2019b). **DoF:** The Dof TFs consists of a bi-functional domain, having dual activity for DNA-binding as well as protein–protein interaction. A single ZF-is present in the C_2_/C_2_ domain needed for binding the target 5′-(T) AAAG-3′ sequence or its reversibly orientated sequence, CTTT, with a conserved region of target DNA sequence. The C-terminal region helps in regulation of the transcription process by interacting with different regulatory proteins. (Yanagisawa, 2002, Noguero et al., 2013). **NF-Y:** NF-YA has two domains with α helix structure. The N-terminal conserved region has 20 amino acids α helix A1 domain, responsible for interaction with NF-YB and NF-YC, while the C-terminal which binds with the CCAAT element has a 21 amino acid α-helix A_2_ domain. NF-YB and NF-YC, is formed through the Histone Fold Domain. These domains bind with each other through head to tail. Subgroups of NF-Y are NF-YA, NF-YB, and NF-YC, binds to the CCAAT box (Petroni et al., 2012, Nardini et al., 2013, Zhao et al., 2017). **HSFs:** Conserved regions of HSFs include three helical structures an N-terminal DBD with four inverted β-sheets arranged in parallel fashion. The binding sites sequence termed heat responsive elements (5′-AGAAnnTTCT-3′) is recognized by the DBD hydrophobic region, which has a helix-turn-helix conformation. At the N-terminal, the oligomeric domain contains two regions of hydrophobic heptapeptide repeats HR-A and HR-B, having five and six heptapeptide repeats, respectively (Yura and Nakahigashi, 1999, Nover et al., 2001, Åkerfelt et al., 2010, Qiao et al., 2015). **Zinc Finger:** Most plants ZF genes have conserved QALGGH amino acid motif within the ZF domain that forms a Q-type C_2_H_2_ plant specific ZF subfamily. This motif is present at the N terminal on an alpha helix. The ZF-motif has zinc, along with two cysteine and two histidine molecules at base, and one alpha helix or two beta-pleated sheets arranged in anti-parallel fashion in a finger like projection. ZFs play role in sub cellular localization and stress responses (Rajavashisth et al., 1989, Iuchi, 2001, Figueiredo et al., 2012, Kaur and Subramanian, 2016).

TFs are classified according to the presence of characteristic sequence motifs which correspond in general to their DNA-binding domains (Fig. 2). Although, there is no strict correlation between sequence type and function, different TF classes tend to have different roles in the hierarchy of responses, although many interact as part of their response, as will be evident from a comparison of the principal classes.

## 3 Role of transcription factors under abiotic stresses

Global warming is becoming an increasing threat to crop productivity as it exposes crops to a plethora of stresses i.e., drought, heat, flooding, salinity, and heavy metals. These environmental factors are menacing crop survival. Abiotic factors affect growth, productivity, and development of plants, and can reduce up to 50% yields of wheat, rice, maize, and cotton (Baillo et al., 2019). Plants respond to stress by certain physiological adjustments i.e., increasing ion fluxes, production of ROS, accumulation of amino acids and soluble sugars, maintaining homeostasis and osmotic potential, and change in phytohormone concentrations (Fig. 1B). The stress-related receptors receive environmental stimuli and activate the stress responsive genes (Leng and Zhao, 2019). The role of different genes/TFs in response to different stresses is discussed below (Table 1).

**Table 1:** Role of different transcriptional factor gene families in abiotic stress tolerance in plants.

Stress	Crop	Transcriptional Factors/Genes	Reference
Drought	*Arabidopsis thaliana*	*AREB1*^↑^*^+^, AREB2/ABF4*^↑^*^+^, ATWRKY1*^↓^*^−^, AtWRKY57*^↑^*^+^, AtWRKY63/ABO3*^↓^*^+^, AtNAC019*^↑^*^+^, AtNAC055*^↑^*^+^, AtNAC072*^↑^*^+^, AtNF-YB7*^↑^*^+^, AtNF-YB3*^↑^*^+^, AtNF-YA5*^↑^*^+^, AtMYB12*^↑^*^+^, AtMYB15*^↑^*^+^, AtMYB33*^↑^*^+^, AtMYB35*^↓^*^+^, AtMYB60*^↓^*^−^, AtMYB44*^↑^*^+^, AtMYB88*^↑^*^+^, AtMYB99*^↓^*^+^, AtMYB96*^↑^*^+^, AtMYB102*^↑^*^+^, AtMYB110*^↓^*^+^*	(Dubos et al., 2010, Tripathi et al., 2014, Feng et al., 2015, Li et al., 2015a, Joshi et al., 2016, Kimotho et al., 2019, Wei et al., 2020)
	*Triticum aestivum*	*TaDREB2*^↑^*^+^, TaDREB3*^↑^*^+^, TaWRKY2*^↑^*^+^, TaWRK19*^↑^*^+^, TaWRKY10*^↑^*^+^, TaNAC69*^↑^*^+^, TaNAC2a*^↑+^*, TaPIMP1*^↑^*^+^, TaMYB1*^↑^*^+^, TaMYB2A*^↑^*^+^, TaMYB19*^↑^*^+^, TaMYB3R1*^↑^*^+^, TaMYB31*^↑^*^+^, TaMYBsdu1*^↑^*^+^, TaMYB30*^↑^*^+^, TaMYB33*^↑^*^+^*	(Dubos et al., 2010, Tripathi et al., 2014, Feng et al., 2015, Li et al., 2015a, Joshi et al., 2016, Li et al., 2016, Baillo et al., 2019, Kimotho et al., 2019, Wei et al., 2020)
	*Oryza sativa*	*OsDREB1F*^↑^*^+^, OsDREB2A*^↑^*^+^, OsEREBP1*^↑^*^+^, OsWRKY01*^↑^*^+^, OsWRKY2*^↑^*^+^, OsWRKY5*^↑^*^+^, OsWRKY7*^↑^*^+^, OsWRKY43*^↑^*^+^, OsWRKY11*^↑^*^+^, OsWRKY45*^↑^*^+^, OsWRKY47*^↑^*^+^, OsNAC10*^↑^*^+^, OsNAC2*^↑^*^+^, OsNF-YA7*^↑^*^+^, OsbZIP42*^↑^*^+^, OsbZIP46*^↑^*^+^, OsbZIP62^+^*^↑^*, OsMYB2*^↑^*^+^, OsMYB4*^↑^*^+^, OsMYB55*^↑^*^+^, MYB59*^↑^*^+^, OsMYB48-1*^↑^*^+^*	(Dubos et al., 2010, Tripathi et al., 2014, Feng et al., 2015, Li et al., 2015a, Joshi et al., 2016, Li et al., 2016, Baillo et al., 2019, Kimotho et al., 2019, Wei et al., 2020)
	*Zea mays*	*ZmDREB1A*^↑^*^+^, ZmDREB2A*^↑^*^+^, ZmDBF3*^↑^*^+^, ZmDREB2.7*^↑^*^+^, ZmWRKY106*^↑^*^+^, ZmNAC55*^↑^*^+^, ZmSNAC1*^↑^*^+^, ZmNAC111*^↑^*^+^, ZmNF-YB2*^↓^*^+^, ZmNF-YA3*^↓^*^+^, ZmHSF14*^↑^*^+^, ZmHSF20*^↑^*^+^, ZmbZIP72*^↑^*^+^, ZmbZIP4*^↑^*^+^, ZmbZIP60*^↑^*^+^, ZmMYB95*^↑^*^+^, ZmMYB36*^↑^*^+^*	(Dubos et al., 2010, Tripathi et al., 2014, Feng et al., 2015, Li et al., 2015a, Joshi et al., 2016, Li et al., 2016, Lan Thi Hoang et al., 2017, Baillo et al., 2019, Kimotho et al., 2019, Li et al., 2020b, Wei et al., 2020)
	*Glycine* max	*GmDREB2A;2*^↑+^*, GmERF3*^↑^*^+^, GmWRKY54*^↑^*^+^, GsWRKY20*^↑^*^+^, GmNAC085*^↑^*^+^, GmNF-YA3*^↑^*^+^, GmMYB177*^↑^*^+^, GmbZIP2*^↑^*^+^*	(Dubos et al., 2010, Tripathi et al., 2014, Feng et al., 2015, Li et al., 2015a, Joshi et al., 2016, Li et al., 2016, Lan Thi Hoang et al., 2017, Baillo et al., 2019, Kimotho et al., 2019, Li et al., 2020b, Wei et al., 2020)
	*Vigna radiate*	*VrDREB2A*^↑^*^+^, VrDREB2B*^↑^*^+^*	(Joshi et al., 2016)
	*Camellia sinensis*	*CsDREB2A*^↑^*^+^, CsDREB2B*^↑^*^+^*	(Joshi et al., 2016)
	*Hordeum vulgare*	*HvWRKY38*^↑^*^+^*	(Tripathi et al., 2014)
	*Solanum lycopersicum*	*SpWRKY1*^↑^*^+^*	(Tripathi et al., 2014)
	*Solanum tuberosum*	*StMYB1R-1*^↑^*^+^*	(Hu et al., 2016)
	*Cicer arietinum*	*CarNAC3*^↑^*^+^*	(Li et al., 2015a)
	*Chrysanthemum*	*CmMYB2*^↑^*^+^*	(Hu et al., 2016)
	*Setaria italic*	*SiNF-YA1*^↑^*^+^, SiNF-YB8*^↑^*^+^*	(Feng et al., 2015, Wei et al., 2020)
	*Cynodon dactylon*	*CdtNF-YC1*^↑^*^+^*	(Feng et al., 2015, Wei et al., 2020)
	*Fagopyrum tataricum*	*FtbZIP5*^↓^*^+^*	(Lan Thi Hoang et al., 2017; Li et al., 2020a)
	*Poncirus trifoliate*	*PtrABF*^↑^*^+^*	(Lan Thi Hoang et al., 2017; Li et al., 2020a)
	*Vitis vinifera*	*VvMYB60*^↑^*^+^*	(Li et al., 2015a, Baillo et al., 2019)
	*Gossypium hirsutum*	*GhirNAC2*^↑^*^+^*	(Shang et al., 2020)
Heat	*Arabidopsis thaliana*	*WRKY39*^↑^*^+^, WRKY46*^↑^*^+^, AtWRKY25*^↓^*^+^, AtWRKY26*^↓^*^+^, AtWRKY33*^↓^*^+^, AtNAC42*^↑^*^+^, AtMYB3*^↑+^*, AtMYB6*^↑+^*, AtMYBL2*^↑^*^+^, AtMYB68*^↑^*^+^,*	(Tripathi et al., 2014, Casaretto et al., 2016, Li et al., 2020a)
	*Oryza sativa*	*OsDREB2B*^↑^*^+^, OsWRKY11*^↑^*^+^, OsNAC063*^↑^*^+^, OsTZF1*^↑^*^+^, OsMYB55*^↑^*^+^*	(Yoshida et al., 2008, Li et al., 2010, Nuruzzaman et al., 2013, Tripathi et al., 2014, Casaretto et al., 2016, Guo et al., 2016, Joshi et al., 2016)
	*Zea mays*	*ZmDREB2A*^↑^*^+^, ZmWRKY106*^↑^*^+^, ZmNF-YA3*^↑^*^+^, ZmHSF14*^↑^*^+^, ZmHSF20*^↑^*^+^, ZmbZIP60*^↑^*^+^, ZmbZIP4*^↑^*^+^, ZmMYB-R1*^↑^*^+^*	(Yoshida et al., 2008, Li et al., 2010, Tripathi et al., 2014, Feng et al., 2015, Guo et al., 2016, Baillo et al., 2019)
	*Glycine* max	*GmHSP70*^↑^*^+^, GmDREB1*^↑^*^+^*	(Kidokoro et al., 2015, Guo et al., 2016)
	*Capsicum*	*CpDREB2*^↑^*^+^*	(Yoshida et al., 2010, Guo et al., 2016)
	*Camellia sinensis*	*CsNAM*^↑^*^+^*	(Nuruzzaman et al., 2013)
	*Gossypium hirsutum*	*GhHSF37*^↑^*^+^, GhHSF24*^↑^*^+^*	(Guo et al., 2016)
	*Capsicum annum*	*CaHSFA2*^↑^*^+^*	(Guo et al., 2016)
	*Malus domestica*	*MdHSFA-9b*^↑^*^+^*	(Guo et al., 2016)
	*Solanum lycopersicum*	*SlHSF01*^↑^*^+^, SlHSFB1*^↑^*^+^, SlHSFA2*^↑^*^+^, SlHSF04*^↑^*^+^, SlHSF16*^↑^*^+^, SlHSF17*^↑^*^+^, SlHSF18*^↑+^	(Guo et al., 2016, Yang et al., 2016)
	*Lycopersicum esculantum*	*LeAN2*^↑^*^+^*	(Casaretto et al., 2016)
Cold	*Arabidopsis thaliana*	*DREB1A*^↑^*^+^, AtZFP1*^↑^*^+^, AtZFP2*^↑^*^+^, AtZF3*^↑^*^+^, AtNAC019*^↑^*^+^, AtMYB14*^↓^*^−^, AtMYB15*^↓^*^−^, AtMYB44*^↑^*^+^, AtMYBC1*^↑^*^+^*	(Baillo et al., 2019, Kimotho et al., 2019)
	*Triticum aestivum*	*TaWRKY19*^↑^*^+^, TaNAC2a*^↑^*^+^, TaNAC4a*^↑^*^+^, TaNAC57*^↑^*^+^, TaMYB2A*^↑^*^+^, TaMYB3R1*^↑^*^+^, TaMYB56-B*^↑^*^+^*	(Dubos et al., 2010, Kim et al., 2016)
	*Oryza sativa*	*OsDREB1A*^↓^*^+^, OsWRKY71*^↓+^*, OsNAC6*^↑^*^+^, OsNAC5*^↑^*^+^, OsNAC04*^↑^*^+^, OsbZIP73*^↑^*^+^, OsMYB2*^↑^*^+^, OsMYB4*^↑^*^+^, OsMYB3R-2*^↑^*^+^, OsMYBS3*^↑^*^+^,*	(Joshi et al., 2016, Kim et al., 2018, Moon et al., 2019, Yubing et al., 2019)
	*Zea mays*	*ZmDREB2A*^↑^*^+^, ZmDBP3*^↑^*^+^, ZmDREB1A*^↑^*^+^, ZmDBF3*^↑^*^+^, ZmSNAC1*^↑^*^+^, ZmNAC55*^↓^*^+^, ZmbZIP60*^↑^*^+^, ZmMYB53*^↑^*^+^, ZmMYB-R1*^↑^*^+^*	(Joshi et al., 2016, Kim et al., 2018, Moon et al., 2019, Yubing et al., 2019)
	*Glycine* max	*GmWRKY21*^↑^*^+^, GmNAC20*^↑^*^+^, GmMYB92*^↑^*^+^, GmbZIP44*^↑^*^+^, GmbZIP62*^↑^*^+^*	(Joshi et al., 2016, Kim et al., 2016, Baillo et al., 2019)
	*Vitis acerifolia*	*VaWRKY12*^↑^*^+^*	(Kim et al., 2018)
	*Pyrus communis*	*PcMYB10*^↑^*^+^*	(Dubos et al., 2010)
Salinity	*Arabidopsis thaliana*	*AtDREB1A/CBF3*^↑^*^+^, AtWRKY25*^↓+^*, AtWRKY33*^↓^*^+^, AtNAC055*^↑^*^+^, AtNAC072*^↑^*^+^, AtNAC019*^↑^*^+^, AtNAC063*^↑^*^+^,ANAC069*^↓^*^−^ AtMYB20*^↑^*^+^, AtMYB41*^↑^*^+^, AtMYB44*^↑^*^+^, AtMYB73*^↓^*^−^, AtMYB88*^↑^*^+^, AtMYB124*^↑^*^+^*	(Golldack et al., 2011, Li et al., 2014, Li et al., 2015a, Baillo et al., 2019)
	*Triticum aestivum*	*TaWRKY2*^↑^*^+^, TaWRKY19*^↑^*^+^, TaNAC2a*^↑^*^+^, TaNAC4a*^↑^*^+^, TaNAC6*^↑^*^+^, TaNAC7*^↑^*^+^, TaMYB1*^↑^*^+^, TaMYB2A*^↑^*^+^, TaMYB3R1*^↑^*^+^, TaMYBsdu1*^↑^*^+^, TaMYB33*^↑^*^+^, TaMYB73*^↑^*^+^*	(Li et al., 2015a, Baillo et al., 2019)
	*Oryza sativa*	*OsDREB1F*^↑^*^+^, OsDREB2A*^↑^*^+^, OsWRKY43*^↑^*^+^, OsWRKY45*^↑^*^+^, OsWRKY5*^↑^*^+^, OsWRKY7*^↑^*^+^, OsWRKY30*^↑^*^−^, OsWRKY72*^↑^*^−^, OsNAC6*^↑^*^+^, OsNC5*^↑^*^+^, OsNAC1*^↑^*^+^, OsNAC063*^↑^*^+^, OsMYB2*^↑^*^+^, OsMYB3R-2*^↑^*^+^, OsMYB91*^↑^*^+^, OsMYB48-1*^↑^*^+^, OsZFP245*^↑^*^+^, OsZFP252*^↑^*^+^, Os ZFP182*^↑^*^+^, OsZFP179*^↑^*^+^*	(Golldack et al., 2011, Li et al., 2014, Li et al., 2015a, Baillo et al., 2019)
	*Zea mays*	*ZmDREB2A*^↑^*^+^, ZmWRKY106*^↑^*^+^, ZmbZIP60*^↑^*^+^, ZmbZIP72*^↑^*^+^, ZmbZIP4*^↑^*^+^, ZmMYB36*^↑+^*, ZmMYB-R1*^↑^*^+^, ZmSNAC1*^↑^*^+^*	(Golldack et al., 2011, Li et al., 2015a, Joshi et al., 2016, Kimotho et al., 2019)
	*Glycine* max	*GmWRKY54*^↑^*^+^, GmWRKY20*^↑^*^+,^ GmWRKY13*^↑−^*, GmNAC20*^↑^*^+^, GmbZIP44*^↑^*^+^, GmbZIP110*^↑^*^+^, GmbZIP62*^↑^*^+^, GmMYB177*^↑^*^+^, GmMYB76*^↑^*^+^, GmMYB92*^↑^*^+^*	(Tripathi et al., 2014, Li et al., 2015a, Joshi et al., 2016, Kimotho et al., 2019)
	*Vigna radiate*	*VrDREB2A*^↑^*^+^*	(Golldack et al., 2011)
	*Camellia sinensis*	*CsDREB2A*^↑^*^+^, CsDREB2B*^↑^*^+^, CsNAM*^↑^*^+^*	(Golldack et al., 2011, Li et al., 2015a)
	*Agrostis stolonifera*	*AsNAC60*^↑^*^+^*	(Golldack et al., 2011, Li et al., 2015a)
	*Setaria italic*	*SiNAC*^↑^*^+^, SiNF-YA1*^↑^*^+^*	(Feng et al., 2015, Li et al., 2015b)
	*Cynodon dactylon*	*CdtNF-YC1*^↑^*^+^*	(Feng et al., 2015)
	*Fagopyrum tataricum*	*FtbZIP5*^↑^*^+^*	(Joshi et al., 2016)
	*Medicago truncatula*	*MtMYB199*^↑^*^+^, MtMYB634*^↑^*^+^, MtMYB636*^↑^*^+^, MtMYB1070*^↑^*^+^*	(Baillo et al., 2019)
	*Gossypium hirsutum*	*GhZFP1*^↑^*^+^*	(Baillo et al., 2019)
Water logging	*Arabidopsis thaliana*	*AtNAC102*^↑^*^+^, AtNAC063*^↑^*^+^, AtAREB1*^↑^*^+^, AtAREB2/ABF4*^↑^*^+^, AtABF3*^↑^*^+^, AtABF2*^↑^*^+^*	(Nuruzzaman et al., 2013)
	*Zea mays*	*ZmEREB180*^↑^*^+^*	(Nuruzzaman et al., 2013)
	*Camellia sinensis*	*CsNAM*^↑^*^+^*	(Nuruzzaman et al., 2013)
	*Oryza sativa*	*OsDREB2A*^↑^*^+^*	(Nuruzzaman et al., 2013)
Heavy Metal Stress	*Arabidopsis thaliana*	*AtMYB48*^↑^*^+^, AtMYB28*^↑^*^+^, AtMYB72*^↑^*^+^, AtMYB124*^↑^*^+^, AtMYB4*^↑^*^+^*	(Jalmi et al., 2018)
	*Zea mays*	*ZmbZIP54*^↑^*^+^*	(Baillo et al., 2019)
	*Glycine* max	*GmbZIP62*^↑^*^+^, GmbZIP44*^↓^*^+^, GmbZIP78*^↓^*^+^*	(Baillo et al., 2019)
	*Triticum aestivum*	*TaHSFA4a*^↑^*^+^*	(Åkerfelt et al., 2010)

Upward arrow (↑) indicates gene upregulation; Downward arrow (↓) indicates gene downregulation; “+” sign indicates positive role of TFs; “-” sign indicates negative role of TFs, under stress conditions.

### 3.1 Drought stress

Drought is a devastating abiotic stress, which occurs due to shortage of ground water, high temperature, and/or low rainfall. Drought stress reduces seedling emergence, germination rate, vegetative growth, root & shoot dry matter, and hypocotyl length (Zeid and Shedeed, 2006). It decreases turgor pressure and limits cell elongation, cell growth, and leaf expansion. Acute shortage of water damages the thylakoid membranes and photosynthetic pigments and reduces the photosynthetic rate. During drought stress, plants close stomata, thereby reducing intracellular CO_2_ concentration thus reducing photosynthesis and also inducing oxidative damage (Fahad et al., 2017). Plants respond to stress by certain physiological adjustments, i.e., increasing ion fluxes, production of ROS, accumulation of amino acids, and soluble sugars and changes in phytohormone levels. TFs play an important role in orchestrating these processes by activating genes that execute stress responses (Leng and Zhao, 2019).

WRKY TFs play an important role in improving stress tolerance, particularly drought and heat in various crop plants. *TaWRKY1* and *TaWRKY33* in *Arabidopsis thaliana* increases drought tolerance due to overexpression of downstream stress responsive genes. *AtWRKY1* locates in the nucleus and binds to the W-box domains of *AtDREB1A*, *AtMYB2*, and *AtAB15* to control their transcription and regulate stomatal conductance (Qiao et al., 2015); in transgenic *Arabidopsis ZmWRKY40*, activated stress related genes and generation of ROS. Overexpression of *TaWRKY2* in wheat increased drought tolerance and grain yield. *Arabidopsis* based *AtWRKY30* was overexpressed in transgenic wheat, resultantly biomass, plant growth, proline concentration, soluble sugar, protein, relative water content, chlorophyll content, and antioxidant enzymes activities were increased to alleviate drought stress (Baillo et al., 2019; Wang et al., 2021). In *Arabidopsis thaliana,* the bZIP gene, *AtABP9* binds to an ABPR motif and increase photosynthetic activity. It increases production of abscisic acid (ABA) and changes composition of photosynthetic pigments. *ZmNF-YB16* overexpressed in young seedling of maize under drought conditions, as a result, antioxidant enzymatic activity was increased to normalize the stress effects (Leng and Zhao, 2019).

Similarly, *SlWRKY8* overexpressed under drought conditions and activated malondialdehyde (MDA), hydrogen peroxide (H_2_O_2_) production, and antioxidant enzymatic activity. These changes triggered stress responsive genes i.e. *SIRD29*, *SIAREB*, and *SIDREB2A* genes, which resulted in decrease stomatal aperture, oxidative pressure, and increases relative water and proline contents to alleviate drought stress. *DREB1A* overexpressed in *Arabidopsis thaliana*, resulted in high accumulation of solutes and initiation of late embryogenesis abundant protein (Kudo et al., 2017). Similarly, soybean *GmNAC8* overexpressed under drought stress and regulated expression of *GmDi19-3* (drought-induced genes), which increased proline and superoxide dismutase (SOD) accumulation. Overexpression of *ZmNAC111* increased water use efficiency of drought prone maize seedling by upregulating drought responsive genes (Yang et al., 2020).

### 3.2 Heat stress

Heat stress adversely affects plant growth by reduced chlorophyll contents and induction of oxidative stress due to accumulation of hydroxyl (OH^−^), hydroperoxyl (HO^2−^), alkoxy (RO^−^), and superoxide (O^2−^) radicals. Oxidative stress hinders photosynthesis and respiratory activities, disrupts protein structure, and membrane integrity (Wassie et al., 2020). Heat stress causes burning of leaves and branches, patch formation on leaves, reduction in germination and growth, reduced tillering, and reduction of grain size and grain yield (Fahad et al., 2017). Elevated temperature uplifts the rate of transpiration, which adversely affects root growth. Sucrose phosphate synthase, adenosine diphosphate-glucose pyro-phosphorylase, and invertase are highly sensitive enzymes that disturb sucrose and starch synthesis pathways during heat stress. On the other hand, high temperature increases catalytic activity of Rubisco, but decreases its ability to bind with CO_2_ and O_2_, and slows down the photosynthetic rate (Crafts-Brandner and Salvucci, 2002).

*Triticum aestivum*, *Solanum lycopersicum*, *Cicer arietinum*, *Glycine* max, and *Sorghum bicolor* are heat-sensitive crops. Similarly, pollens formation, seed setting, and grain filling are highly heat- sensitive plant stages (Hinojosa et al., 2019). Plants have several adaptations against heat stress, i.e., degradation of oxyradicals, reduction in the lipid membrane transformation stage, and biological metabolism. Epigenetic modifications, i.e., acetylation, methylation, phosphorylation, and ribosylation also play role in plant survival during heat stress by modifying histone proteins after translation (Hou et al., 2019). Evolution of heat shock factors (HSFs) is another modification of plants in response to heat stress. *HsfA1s* are prime activators in response to heat stress, while in non-stress conditions; these are suppressed by heat shock proteins, i.e., HSP70 and HSP90. These regulate expression of some TFs, i.e., dehydration responsive element binding 2A (*DREB2A*), heat shock factors A2 (*HsfA2*), heat shock factor B (*HsfBs*), *DREB2C*, multiprotein binding factor 1C (*MBF1C*), and NAC. HSPs are involved in homeostasis at the cellular level and plant defence. At the onset of heat stress, inactive *HSFs* are activated through oligomerization and shuttle signalling between the cytoplasm and nucleus (Lohani et al., 2019).

HSP70 makes a complex with heat stress *RNA1* (*HSR1*) and translation elongation factor (*eEF1A*), which activates *HSF1* which in turn activates the cell heat stress responsive machinery (Rangan et al., 2020). A complex of TFs network consisting of MYB, bZIP, NAC, and a homeobox linked with Leucine zipper is recognized as effective elements in long-term heat stress conditions. General heat stress responsive elements and stabilizers for protein metabolism are *HSP10s*, *HSP20s*, *HSP60s*, *HSP90s*, and co-chaperones (Jung et al., 2012). Different WRKY i.e., *AtWRKY18*, *AtWRKY25*, *AtWRKY33*, *AtWRKY40*, and *AtWRKY46* also play vital role during heat stress. *AtWRKY39* imparts heat stress tolerance in *Arabidopsis thaliana* by binding to a calmodulin binding TF (Li et al., 2010). The *AtDREB2A* bind to dehydration responsive elements (DRE) at the promoter site of *AtHSFA3*, and activates a stress response through an ABA-independent pathway (Yoshida et al., 2008).

### 3.3 Salinity stress

Globally, around 30% of arable land is affected by salt stress and the proportions continue to increase at a rapid pace due to driving force of urbanization. Salt stress inhibits imbibition, decreases root elongation and germination percentage (Kaymakanova, 2009). Salinity and drought are sister stresses, plants face drought stress in media or soil affected by salt stress (Shahbaz and Ashraf, 2008). Osmotic stress closes stomata, decreases photosynthetic rate, and disrupts action of the thylakoid membrane or Calvin cycle enzymes (Hussain and Reigosa, 2015). It alters leaf anatomy, i.e., thickness of epidermis, mesophyll, palisade length, and diameter. Plants start producing ROS (O^2−^ ion, H_2_O_2_ and OH^−^) in chloroplast, cytosol, apoplastic space, and mitochondria. Activation of ROS results in oxidation of carbohydrates, lipids, proteins, nucleic acid, and impacts membrane integrity. OH^−^ ion causes damage to DNA by disrupting purine and pyrimidine (Shahzad et al., 2019).

Plants maintain homeostasis within and outside the cytoplasm for normal growth (Hasegawa, 2013). Channel proteins, anti porters and symporters, maintain ion transport during homeostasis. Moreover, compatible osmolytes, i.e., free amino acid sugars, quaternary ammonium compounds, and proline are produced (Ashrafijou et al., 2010). These osmolytes protects cell structure and maintain osmotic balance by continuous water flux. Glycine betaine, an organic compound, plays a significant role in lowering salt stress by osmotic adjustment, protecting photosynthetic machinery, and protein stabilization. Various antioxidant enzymes, such as superoxide dismutase (SOD), ascorbate peroxidase (APX), glutathione reductase (GR), glutathione peroxidase (GPX), and non-enzymatic antioxidants, such as carotenoids and tocopherols, act as scavengers against ROS (Shahzad et al., 2019). All these events are regulated by large number of salt responsive genes which are governed by TFs, which percept signal and start defense mechanism (Ciarmiello et al., 2014).

Three types of genes involved in sensing and signaling stress, transport regulators, and salt stress-response-related genes, play key role under salt stress. When stress occurs, Na^+^ ion enters the cell via non-selective cation channels and other membrane transporters. These Na^+^ ions are recognized by unknown sensory elements. In the second step, ROS, Ca^2+^, and other hormones act as secondary messengers against salt stress and induce altered transcriptomic profile (Amirbakhtiar et al., 2019). Some common examples of role of transcriptional factors in response to salt stress are illustrated below. *AtNAC2,* a nuclear localized gene, is upregulated during salt stress and promoted lateral root development (He et al., 2005). Over-expression of *AtWRKY46* increase lateral root development through an ABA signaling pathway. Overexpression of *Gossypium hirsutum* derived *GhWRKY34* in *Arabidopsis* increased salt tolerance by inducing selective uptake of Na^+^ or K^+^ ions in roots and leaves (Finatto et al., 2018). *Reaumuria trigyna* derived *RtWRKY1* overexpressed under salt stress and resulted in increased root growth, anti-oxidative enzymes and decreased Na^+^ or Na^+^/K^+^ ratio (Du et al., 2017). Overexpression of *GmWRKY54* alleviated salt stress in soybean (Zhou et al., 2008). *AtbZIP24* increased salt tolerance by osmotic balance, ion homeostasis and increased growth and development, involving homo- and heterodimerization, or post-transcriptional modification (Yang et al., 2009). Overexpression of *GmERF3* in tobacco increased free proline and soluble carbohydrates and relieved salt stress.

### 3.4 Cold stress

Cold stress affects cellular metabolism by decreasing the rate of biochemical reaction, concentration of nucleic acid, and protein. Plants respond to cold stress by increasing proline contents, membrane fluidity, and ROS activation (Zheng et al., 2019). ROS causes oxidative damage in cells, reduced enzymatic activity, cause ionic imbalance, damaged cell membranes, reduced respiration rate, and degrade proteins. Plants also face low germination, delayed cell cycle, low leaf development rate, decreased seedling vigor, and yield (Hussain et al., 2018). Root development is also affected, resulting in lowered biomass and root length, and reduced root volume. Onset of cold stress at the reproductive stage leads towards pollen sterility, abortion of flower or ovules, distortion of pollen tubes, poor fruit setting, deformation of panicles, spikelet degeneration, and reduced productive tillers (Li et al., 2015a). The photosynthetic rate decreases due to reduced CO_2_ conductance in the mesophyll and stomata, restricted transport of metabolites and increased photo-inhibition (Hussain et al., 2018).

Different TFs respond to cold stress by regulating expression of cold responsive genes i.e. dehydrin genes, abscisic acid responsive genes, and late embryogenesis abundant genes. These TFs are present in the nucleus throughout as they possess nucleus-localization signals (NLSs). Under stress, membrane bounded TFs are activated, enter the nucleus, and regulate gene expression. Some of the highlighted examples are illustrated here. Nuclear-localized *SlNAC1* activate stress responsive genes and enhances cold tolerance. *ZmSNAC1*, *OsNAC5,* and *TaNAC57* genes are overexpressed and enhance cold tolerance (Li et al., 2014). *Arabidopsis thaliana* and grapevine calli showed overexpression of *VaWRKY12* and affected downstream located genes encoding antioxidant enzymes, glutathione S-transferases, and peroxidase in response to cold stress (Zhang et al., 2019). *OsWRKY71* was upregulated in rice under cold stress. Overexpression of *CsWRKY* enhanced cold tolerance by affecting root development, germination rate, seed development, flowering, and dormancy in cucumber (Kim et al., 2016). *OsbZIP73* upregulated and played an important role at flowering, seedling, and reproductive stages to counter cold stress. It co-expressed with *OsbZIP71* and enhanced seed set by affecting pollen fertility through an increased production of soluble sugar in pollen and decreased accumulation of ABA in anthers (Liu et al., 2019).

*OsbZIP87* and *OsbZIP38* also played an important role in enhancing cold tolerance in rice (Liu et al., 2019). *OsDREB1G* regulates expression of cold induced genes present in protoplasts. *Arabidopsis thaliana* based TFs, *AtDREB1A/CBF1* and *AtCBF4* are overexpressed in response to cold stress (Moon et al., 2019). MYBs also play vital role in combating cold stress by affecting cell cycle, cellular morphogenesis, hormonal signaling, secondary metabolism, and gene expression. *AtMYB14* was down regulated under cold stress and encoded the proteins that act as the R2R3-MYB activator. As a result, CBF proteins (*CBF1*, *CBF2*, and *CBF3*) are activated, which initiate cold responsive genes. In transgenic *Arabidopsis thaliana GmMYBJ1* was overexpressed and enhanced cold tolerance. *GmMYBJ1* regulated expression of many stress responsive genes i.e. *AtCOR15a*, *AtRD29B*, *AtP5CS,* and *AttCOR78* increased cold tolerance. *OsMYB4* in *Arabidopsis thaliana* was also overexpressed and increased freezing tolerance (Su et al., 2014).

### 3.5 Heavy metal stress (HMS)

Heavy metals (HMs) are generally defined as metals with relatively high densities, atomic weights, or atomic numbers, i.e., Mo, Ni, Cd, Cr, and Zn. *HMs* enriched soil results in reduced growth, altered nutrients and water balance, inhibition of photosynthesis and chlorosis of crop plants. Further, *HMs* affect chlorophyll content, photosystem II effectiveness, and Rubisco activity (Maleva et al., 2012). These decreases reductant pool as a result less ATPs are produced and less CO_2_ is fixed (Singh et al., 2016). Heavy metals enhances protease activity decreases ammonia glutamate dehydrogenase (GDH), glutamine oxoglutarate aminotransferase (GOGAT), glutamine synthetase (GS), nitrite reductase (NiR), and nitrate reductase (NR) activity (Chaffei, 2003). ROS accumulation, cause oxidative stress by interacting with proteins, DNA, and lipids, and destabilizing cellular organization (Sharma et al., 2012).

Cd stimulates the myelin basic protein (MBP) kinase gene and *OsMAPK2* in *Oryza sativa*. Due to heavy metal stress, different mitogen activated protein kinases (MAPKs) are activated. *Oryza sativa multiple stress responsive MK2* (*OsMSRMK2*), *wound and JA-uninducible MK1* (*OsWJUMK1*), and *OsMSRMK3* are induced in response to Cd^2+^ and Cu^2+^ in roots and leaves (Ali et al., 2019). Similarly, *Stress Activated MAPK* (*SAMK*), *Medicago MAP kinase3* (*MMK_3_*), *Medicago MAP kinase2* (*MMK_2_*), and *salt stress induced MAPK* (*SIMK*) are activated in *Medicago sativa* in response to CdCl_2_ and CuCl_2_ stress (Opdenakker et al., 2012b). Downstream TFs i.e., activator protein 2 (AP_2_), WRKY, bZIP, MYB, DREB, ERF, and ZAT (C_2_H_2_ type ZF-) are stimulated by activation of MAPKs as their targets. Likewise, *Arabidopsis thaliana*, in response to CuSO_4_ and CdCl_2_ stress, starts accumulation of MPK_3_ and MPK_6._ Moreover, different TFs i.e. *AtMYB28*, *AtMYB72*, *AtMYB48*, *AtMYB124,* and *AtMYB4* are upregulated in *Arabidopsis* plants subjected to Cd and Zn stress. A short duration exposure of *Arabidopsis* roots to Cu stress activated *AtWRKY22*, *AtWRKY25,* and *AtWRKY29,* whereas long term exposure activated *AtWRKY25* and *AtWRKY29* (Opdenakker et al., 2012a). In *Arabidopsis thaliana*, Cd stress upregulated ethylene responsive factors i.e., *AtERF1*, *AtERF5,* and *AtZAT6* to manipulate plant metabolism for stress tolerance (Jalmi et al., 2018). Similarly, in *Glycine* max*,* up-regulation of *GmbZIP62* and down-regulation of *GmbZIP44* and *GmbZIP78* was observed under Cd stress (Hong et al., 2017).

### 3.6 Waterlogging stress

Water logging is a combination of two important stresses: submergence (when the whole plant is under water) and water logging (when only roots are submerged) (Zhao et al., 2018). It results from prolonged rainfall, poor drainage of soil, and its intensity tends to increase every year (Sundgren et al., 2018). Diffusion of gases in water is low in comparison to air, which give rise to oxidative stress (Colmer and Greenway, 2011). Different physiological and biochemical processes are activated, which shift aerobic respiration to anaerobic fermentation. Moreover, certain toxic compounds, such as alcohol and aldehydes are accumulated in the cytoplasm (Zhou et al., 2020). There are two stages of water logging, hypoxia (partial depletion of oxygen) and anoxia (complete depletion of oxygen). Different plant processes, i.e., cytoplasmic pH, cellular energy, stem elongation, and adventitious root formation decreases. In addition, fresh and dry mass decreases; and the electron-transport chain and CO_2_ assimilation are also affected (Barickman et al., 2019). Furthermore, toxic compounds and ROS accumulated. Certain redox enzymes, such as cupredoxins, are activated to maintain ROS balance (Jin et al., 2017).

Roots also play an important role in response to water logging stress, as aerenchyma and adventitious roots are established. The function of aerenchyma is to increase internal diffusion of oxygen from aerial parts to waterlogged roots to facilitate an aerobic environment (Fukao et al., 2019). The known transcription factors that play roles in regulation of water logging stress include bZIP, NAC, WRKY, MYB, and ERFs. But the highest number of transcription factors responding to waterlogging stress belong to the MYB and the AP2/EREBP gene families (Borrego-Benjumea et al., 2020). Low oxygen-induced genes are characterized by an anaerobic response element (ARE) present in the promoter. ARE has GC and the GT motifs, which are important for gene activity and signal transduction (Dennis et al., 2000). In *Arabidopsis,* five ERF VII genes *AtHRE1*, *AtHRE2*, *AtRAP2.2*, *AtRAP2.3*, and *AtRAP2.12* played significant role under waterlogging conditions. Rice *SUB1A* is considered a master regulator against water logging stress. These ERF VII tandem repeats are responsible for increasing inter-nodal elongation and enable the plants to overcome waterlogged conditions (Fukao et al., 2019). *ZmEREB180* increased waterlogging tolerance in maize seedlings due to conserved N terminal motif and its ectopic expression (Yu et al., 2019). Overexpression of *AtSHYG* (*AtNAC047*) caused hyponastic growth in *Arabidopsis* (Hofmann, 2013). Moreover, *SiWRKY51* and *SiWRKY65* also play important roles in roots of waterlogged plants (Li et al., 2017). However, exploring the role of TFs under waterlogging stress needs further attention.

## 4 Role of transcriptional factors under biotic stresses

Biotic stresses such as diseases, insects, and nematodes adversely affect plant growth, development, survival, and crop productivity. Reported losses due to biotic stresses are up to 35% (Baillo et al., 2019). Yield losses in USA due to *Fusarium* head blight and wheat rust amounted to US $3 billion and US $5 billion, respectively. In the middle of the 19th century, during the Irish potato famine, the crop were completely destroyed due to late blight (*Phytophthora infestans*), leaving millions of farmers empty handed (Rashad and Moussa, 2020). The available data suggests that a decrease in annual crop productivity by arthropods is 18–20% worldwide, amounting to US $470 billion. The most prone area’s to biotic stresses are African and Asiatic countries (Sharma et al., 2017). Diseases negatively affects morphological characteristics, i.e., plant height, chlorophyll content, and leaf architecture (Cerda et al., 2017).

To deal with these challenges, plant adopt coordination of different physiological, biochemical, and molecular processes through signal transduction mechanisms (Amorim et al., 2017). Pathogen attack signals are recognized through pathogen-associated molecular patterns (PAMPs) present on the host surfaces that trigger a basic immune response PAMP-triggered immunity (PTI). Plants have resistance proteins (R) that directly or indirectly identify effectors and activate effector-triggered immunity (ETI), such as hypersensitive response (HR). The detailed infection process of bacterial, fungal, and viral pathogens, insects, and nematodes, is described below, along with a variety of defense responses for each infection.

### 4.1 Bacterial infections

More than 200 pathogenic bacterial species have been identified in plants. The most important bacterial infections belongs to genera *Pseudomonas*, *Ralstonia*, *Agrobacterium*, *Xanthomonas*, *Erwinia*, *Xylella*, *Pectobacterium,* and *Dickeya*. Pathogenic bacteria produce cell wall degrading enzymes, which provide passage for infiltration and maceration in plant tissue for feeding. *Erwinia amylovora* causes fire blight of the Rosaceae family, apple, and pear (Mansfield et al., 2012). *Ralstonia solanacearum* causes bacterial wilt of tomato, tobacco, banana, and the brown rot of potato. *Xanthomonas* mainly effect rice, banana, tomato, and citrus fruits, and invades mostly xylem or parenchyma tissues (Ryan et al., 2011). *Xylella fastidiosa* is a xylem-limited phytopathogen, which causes diseases in grapes, almond, citrus, peach, coffee, and olive trees. *Pseudomonas syringea* causes infection in the tomato by forming a necrotic lesion surrounded by yellow chlorotic halo on the tomato (termed as bacterial speck) (Buttimer et al., 2017).

After a pathogen attack, TFs activate pathogen related (PR) genes and promote HR. HR is responsible for tissue necrosis by systemic acquired resistance (SAR). Defense-related TFs include bZIP, AP_2_/ERF, NAC, MYB, DOF, and WRKY, which play an important role in defense response against pathogen attacks. Some examples pertaining to role of TFs in response to bacterial disease are explained below however detailed information is summarized in the Table 2. *OsWRKY80* and *OsWRKY4* genes incorporate resistance against rice sheath blight. *OsWRKY80* attached to a W-box in the promoter region of *OsWRKY4* and activated defense response against *Rhizoctonia solani*. Moreover, *OsWRKY7*, *OsWRKY58*, *OsWRKY64*, and *OsWRKY76* are also upregulated in the rice plants subjected to rice blast (Baillo et al., 2019). Overexpression of *VaERF20* increased resistance against *Pseudomonas syringae* and *Botrytis cinerea* in transgenic *Arabidopsis* (Wang et al., 2018a). *SlCabZIP* and *SlERF11* eliminated pathogenicity of *Pseudomonas syringae* Pv. tomato DC3000 and provide resistance (Lee et al., 2003). *AtNAC032* repressed activation of MYC on pest attack by blocking a coronatine mediated reopening of stomata and thus stopped the entry of *Pseudomonas syringae* Pv. tomato DC3000 (Allu et al., 2016). *GhWRKY39-1* provides resistance to Root rot (*R. solani*) in *Gossypium hirsutum* (Chen et al., 2017). *CaWRKY27*, *CaWRKY6* mediate bacterial wilt while *CaWRKY58* resisted bacterial spot (*Xanthomonas axonopodis*) (Erpen et al., 2018). *SINAC35* counter bacterial wilt (*R. solanacearum*) and bacterial spot (*X. compestris*) infections in *Capsicum annuum* (Baillo et al., 2019), as explained in Table 2.

**Table 2:** Role of different transcriptional factor gene families in biotic stress tolerance in plants.

**Stress**	**Crop**	**Disease**	**Gene**	**Reference**
Bacterial	*Arabidopsis thaliana*	Bacterial Leaf spot *(Pseudomonas syringae)*	*AtWRKY22*^+↓^*, AtWRKY29*^+↓^*, AtWRKY38*^−↑^*, AtWRKY41*^+↑^*, AtWRKY62*^−↑^*, AtERF014*^+↑^*., AtNAC19*^+↑^*, AtNAC55*^+↑^*, AtNAC72*^+↑^*, AtMYB30*^+↑^*, AtMTB44*^+↑^*, AtMYB96*^+↑^_,_*AtNAC042/JUB1*^−↑^_,_*CBNAC/NTL9*^−↑^_,_	(Chen et al., 2017, Erpen et al., 2018, Baillo et al., 2019, Yuan et al., 2019b)
	*Vitis vinifera*	Bacterial Leaf Spot (*Pseudomonas syringae*)	*VvERF20*^+↑^	(Chen et al., 2017, Erpen et al., 2018)
	*Oryza sativa*	Bacterial Blight (*Xanthomonas oryzae*)	*OsWRKY6*^+↑^*, OsWRKY45*^+↑^*OsWRKY67*^+↑^*, OsNAC58*^+↑^*, OsNAC66*^+↑^*, OsWRKY13*^+↑^*, OsWRKY71*^+↑^*, OsEREBP1*^+↑^*.*	(Chen et al., 2017, Erpen et al., 2018, Baillo et al., 2019, Yuan et al., 2019b)
	*Capsicum annuum*	Bacterial Wilt (*Ralstonia solanacearum*)	*CaWRKY27*^+↑^*, CaWRKY6*^+↑^*,*	(Erpen et al., 2018)
		Bacterial Spot (*Xanthomonas axonopodis*)	*CaWRKY58*^−↑^	(Erpen et al., 2018)
		Pepper Root Rot (*Bacillus thuringiensis*)	*CaPF1*^+↑^	(Chen et al., 2017)
	*Solanum lycopersicum*	Bacterial Wilt (*Ralstonia solanacearum*)	*SlERF3*^+↑^*, SlERF5*^+↑^***,****SlNAC35*^+↑^	(Erpen et al., 2018)
		Bacterial Spot (*Xanthomonas campestris*)	*SlERF1*^+↑^***,****SlNAC35*^+↑^	(Erpen et al., 2018)
	*Glycine* max	Bacterial Wilt (*Ralstonia solanacearum*)	*GmERF3*^+↑^	(Erpen et al., 2018)
	*Nicotiana tabacum*	Bacterial wilt *(Ralstonia solanacearum)*	*NtWRKY50*^+↑^	(Chen et al., 2017)
	*Manihot esculenta*	Bacterial Blight (*Xanthomonas axonopodis*)	MebZIP3^+↑^, MebZIP5^+↑^	(Erpen et al., 2018)
Fungal	*Arabidopsis thaliana*	Gray mold *(Botrytis cinerea)*	*AtERF1*^+,↑^*, AtERF14*^+,↑^	(Baillo et al., 2019)
		Fusarium wilt *(Fusariuum oxysporum)*	*AtERF2*^+,↑^*, AtERF4*^−↓^	(Baillo et al., 2019)
		Powdery Mildew (*Erysiphe cruciferarum*)	*AtbZIP10*^+↑^	(Erpen et al., 2018)
	*Triticum aestivum*	Yellow Rust *(Puccinia striiformis)*	*TaWRKY49*^+↓^*, TaWRKY62*^+↑^*, TaWRKY70*^+↑^*, TaNAC1*^−↑^*, TaNAC4*^+↑^*, TaNAC8*^+↑^*, TaNAC21/22*^−↑^*, TabZIP74*^+ ↑^, *TaNAC30*^−↑^	(Erpen et al., 2018, Wang et al., 2018b)
		Leaf rust (*Puccinia triticina*)	*TaWRKY1B*^+^	(Kumar et al., 2018)
		Powdery mildew *(Erysiphe cruciferarum)*	*TaNAC6*^+ ↑^*, TaNAC21/22*^−↑^, *TaNAC30*^+↓^	(Yuan et al., 2019a)
		*Root Rot (Bipolaris sorokiniana)*	*TaERF3*^− ↓^*, TaPIEP1*^+↑^	(Baillo et al., 2019)
		*(Rhizoctonia cerealis)*	*TaRIM1*^+↑^	(Baillo et al., 2019)
	*Oryza sativa*	Sheath blight (*Rhizoctonia solani*)	*OsWRKY4*^+↑^*, OsWRKY80*^+↑^	(Erpen et al., 2018)
		Rice Blast (*Magnaporthe oryzae, Pyricularia oryzae*)	*OsWRKY7*^+↑^*, OsWRKY45*^+↑^*, OsWRKY58*^+↑^*, OsWRKY62*^+↑^*, OsWRKY64*^+↑^*, OsWRKY76*^+↑^*, OsWRKY22*^+↑^*, OsNAC6*^+↑^*, OsNAC19*^+↑^*, OsNAC66*^+↑^*, OsNAC122*^+↑^*, OsNAC131*^+↑^	(Erpen et al., 2018, Tolosa and Zhang, 2020)
	*Gossypium hirsutum*	Sheath blight (*Rhizoctonia solani)*	*GhWRKY39-1*^+↑^	(Erpen et al., 2018)
	*Brachpodium distachyon*	Fusarium head blight (*Fusarium graminearum*)	*BdWRKY8*^+↑^*, BdWRKY34*^+↑^*, BdWRKY50*^+↑^*, BdWRKY70*^+↑^*, BdWRKY69*^+↑^	(Erpen et al., 2018)
	Solanum lycopersicum	Gray Mold (*Botrytis cinerea*)	*SlSRN1*^−↓^	(Yuan et al., 2019a)
		Tomato Wilt (*Plectosphearella cucumerina)*	*SlERF1*^+↑^	(Baillo et al., 2019)
		Rhizopus Soft Rot (*Rhizopus nigricans*)	*SlERF1*^+↑^	(Baillo et al., 2019)
	*Saccharum officinarum*	Red Rot (*Colletotrichum falcatum*)	*SobZIP4*^+↑^*, SobZIP15*^+↓^*, SoNACH*^+↓^	(Muthiah et al., 2013)
	*Solanum tubersum*	Late Blight (*Phytophthora infestans*)	*StNAC4*^+↑^*, StNAC5*^+↑^*, StNAC18*^+↑^*, StNAC48*^+↑^*, StNAC81*^+↑^*, StERF3*^−↑^	(Baillo et al., 2019, Tolosa and Zhang, 2020)
	*Hordium vulgare*	Powdery mildew (*Blumeria gramini*)	*HvWRKY10*^+↑^*, HvWRKY19*^+↑^*, HvWRKY28*^+↑^*, HvNAC6*^−↓^	(Erpen et al., 2018)
		Spot Blotch (*Bipolaris sorokiniana*)	*HvMYB6*^+↑^	(Baillo et al., 2019)
	*Glycine* max	Root Rot (Phytophthora sojae)	*GmERF5*^+↑^*, GmERF113*^+↑^	(Baillo et al., 2019)
		Soybean Rust *(Phakospora pachyrhizi*)	*GmbZIP1*^+↑^*, GmbZIP2*^+↑^*, GmbZIP62*^+↑^*, GmbZIP105*^+↑^	(Baillo et al., 2019)
	*Nicotiana benthamiana*	Anthracnose (*Colletotrichum orbicular*)	*NbWRKY8*^+↓^	(Erpen et al., 2018)
	*Vitis vinifera*	Grey Mold *(Botrytis cinerea*)	*VvERF20*^+↑^	(Baillo et al., 2019)
	*Populus trichocarpa*	Popular leaf Rust *(Melampsora medusae)*	*PtrWRKY18*^+↑^*, PtrWRKY35*^+↑^*, PtrWRKY89*^+↑^	(Erpen et al., 2018)
Viral	*Arabidopsis thaliana*	Tobacco mosaic virus (TMV)	*AtWRKY8*^+,↑^*, AtWRKY61*^+,↑^*, ATAF2*^+,↑^	(Chen et al., 2017, Erpen et al., 2018)
	*Nicotiana tobacam*	Tobacco mosaic virus (TMV)	*WRKY8*^+,↑^*, NtERF5*^+,↑^	(Chen et al., 2017, Erpen et al., 2018)
	*Oryza sativa*	Rice Dwarf Virus (RDV)	*OsNAC*^+,↑^	(Yuan et al., 2019a)
		Rice Stripe Mosaic Virus (RSMV)	*OsMYB4*^+,↑^	(Erpen et al., 2018)
	*Solanum lycopersicum*	Tomato Yellow Leaf Curl Virus (TYLCV)	*SlNAC20*^+,↑^*, SlNAC24*^+,↑^*, SlNAC47*^+,↑^*, SlNAC61*^+,↑^	(Yuan et al., 2019a)
Nematodes	*Arabidopsis thaliana*	Cyst nematode (*Heterodera schachtii*)	*AtWRKY23*^+,↓^*, AtMYB12*^+,↑^*, AtWRKY6*^+,↓^*, At WRKY11*^+,↓^*, AtWRKY17*^+,↓^*and AtWRKY33*^+,↓^. *(Downregulation)*	(Hamamouch et al., 2020)
		Root-Knot Nematodes (*Meloidogyne incognita*)	*AtMYB12*^+,↓^	(Hamamouch et al., 2020)
	*Solanum lycopersicum*	Root Knot Nematode (*Meloidogyne javanica)*	*SlWRKY45*^−,↑^*, SlWRKY3*^+,↑^*, SIWRKY70*^+,↓^	(Chinnapandi et al., 2017, Chinnapandi et al., 2019)
	*Glycine Max*	Soybean Cyst Nemadtode (*Heterodera glycines*)	*GmWRKY136*^+,↑^*, GmWRKY53*^+,↑^*, GmWRKY86*^+,↑^	(Yang et al., 2017b)
Insects	*Arabidopsis thaliana*	*Cabbage moth (Pieris brassicae)*	*AtMYB75*^+,↑^	(Shen et al., 2018)
	*Triticum aestivum*	English grain aphid (*Sitobion avenae)*	*TaMYB19*^+,↓^*, TaMYB2*^+,↓^*, TaMYB44^+,^*^↓^	(Shen et al., 2018)
		Russian wheat aphid *(Diuraphis noxia)*	*TaWRKY53*^+,↓^	(Van Eck et al., 2014)
	*Oryza sativa*	Brown plant hopper (*Nilaparvata lugens)*	*OsWRKY45*^+,↓^	(Huang et al., 2016)
		Striped stem borer *(Chilo suppressalis)*	*OsWRKY53*^+,↓^	(Hu et al., 2016)
			*OsERF3*^+,↑^	(Lu et al., 2011)
	*Chrysanthemum*	Aphid *(Aphidodea)*	*CmMYB15^+,^*^↑^*, CmMYB19*^+,↑^	(An et al., 2019)

Upwrd arrow (↑) indicates gene upregulation; Downward arrow (↓) indicates gene downregulation; “+” sign indicates positive role of TFs; “-” sign indicates negative role of TFs, under stress conditions.

### 4.2 Fungal diseases

Economically important fungal diseases are yellow rust, leaf rust, stem rust, spot rust, red rot, sheath blight, rice blast, powdery mildew, downy mildew, and stem canker. Fungal infestation prevents closing of the stomata, damages the xylem cells, disrupt the cuticle layer, causes extensive water loss, decreases leaf and shoot water potential, decrease fresh weight, root number, and length, produces large numbers of brown roots, and reduces uptake and availability of nutrients (Pandey et al., 2017, Jamil et al., 2020a). When plants are subject to fungal attacks, they produce plant hormones, i.e., ethylene, salicylic acid, and jasmonic acid. Plant hormones activates expression of TFs, i.e., AP2/ERF, WRKY, NAC, MYB, and MYC (Luo et al., 2019). The following section covers some of the key examples of role of TFs in resisting fungal pathogens in plants. Overexpression of *AtWRKY72* enhanced resistance against powdery mildew in *Arabidopsis*. Similarly, *AtWRKY8* and *AtWRKY28* enhanced resistance against *Botrytis cinerea*. *TaWRKY49*, *TaWRKY62,* and *TaWRKY70* combat strip rust (*Puccinia striiformis*) by activating ROS, jasmonic acid, salicylic acid, and ethylene production (Chen et al., 2017).

In rice, overexpression of *OsWRKY45* and *OsWRKY22* enhanced resistance to *Pyricularia oryzae*. *OsWRKY45* overexpressed and enhanced resistance against fungal pathogen *Magnaporthe grisea*. *OsWRKY4* and *OsWRKY80* increase sheath blight resistance in rice. *OsWRKY80* binds to the W-box in the promotor region of *OsWRKY4* and works as a positive regulator for *Rhizoctonia solani* resistance. In *Brachpodium distachyon, BdWRKY8*, *BdWRKY50*, *BdWRKY34,* and *BdWRKY70* were upregulated and enhanced resistance against *Fusarium graminearum*. In *Hordeum vulgare, HvWRKY1* cooperated with *HvMYB6* to counter powdery mildew (Jiang et al., 2017). Similarly, *TaNAC6* overexpressed and enhanced powdery mildew resistance and decreased fungal haustoria. *OsNAC6* shows overexpression and enhanced rice blast resistance. In barley, *HvNAC6* overexpressed under powdery mildew infection and increased resistance against *Blumeria gramini*. *VaERF20* increased resistance against *Botrytis cinerea* in transgenic *Arabidopsis*. In soybean, *GmbZIP1*, *GmbZIP62*, *GmbZIP105,* and *GmbZIP2* genes prevented infestation of Asian soybean rust (Baillo et al., 2019).

### 4.3 Viral diseases

Viruses cause a variety of plant diseases. The main symptoms of all diseases are decreased internodal distance, deficiency of chlorophyll, and reduction in growth. Other related symptoms are reduced germination rate, rolled leaf blade, less nodulation, swelling of stem, tumors on stem, roots and leaves, reduced pollen fertility, reduced seed set, wilting, and cell death (Matthews, 2012). Economically important viruses are sorghum mosaic virus (SrMV), sugarcane mosaic virus (SCMV), and sugarcane streak mosaic virus (SCSMV). SrMV and SCMV are effective pathogens for *Sorghum bicolor*, gramineous plants and *Zea mays* (Ling et al., 2018). Other less virulent viruses are yellow vein mosaic virus in okra, urd bean leaf crinkle virus, strawberry mild yellow edge virus, rice stripe mosaic virus, cotton leaf curl virus, sugarcane yellow leaf virus, barley yellow dwarf virus, and maize chlorotic mottle virus. Rapid mutations of viral strains is a major factor behind failure of breeding programs (Jamir et al., 2020). Plant activate hormonal responses, gene silencing, metabolic regulation, cellular protein degradation by the ubiquitin proteasome pathway (UPS), signaling of immune receptors and PAMP-triggered immunity to stop replication of viruses. Accumulation of ROS and plant hormones, i.e., salicylic acid, jasmonic acid, abscisic acid, brassinosteroids, cytokinin, auxin, ethylene, and gibberellin play role in plant defense against viruses (Calil and Fontes, 2017).

Different TFs play significant roles in resisting virus-induced damage as illustrated in the following examples and Table 2. Overexpression of *OsMYB4* is responsible for resistance against viral diseases. The *MtWRKY* gene of *Medicago truncatula* provides resistance against tobacco mosaic virus in *Nicotiana tabacum*. The *Gossypium hirsutum* based *GhWRKY15* gene, when introduced in *Nicotiana tabacum,* showed activity against the tobacco mosaic virus (Erpen et al., 2018). NAC TFs play vital roles in plant immunity by specific signals and virulence action of pathogenic effectors. Viral infection proteins sometime hijack NAC TFs to enable viral replication and decrease host immunity. However, some examples of NAC TFs imparting resistance are as follows: *SlNAC20*, *SlNAC24*, *SlNAC41,* and *SlNAC61* played a significant role in imparting resistance against Tomato Yellow Leaf Curl Virus (TYLCV) (Huang et al., 2017). *Triticum aestivum* NAC TFs, i.e., *AtGRAB1* (Geminivirus Rep A-Binding) and *AtGRAB2*, interacted with Wheat Dwarf Geminivirus (WDV) Rep A protein and hinder DNA replication of WDV. *Arabidopsis thaliana AtAF2* interact with the Tobacco Mosaic Virus (TMV) helicase domain and its overexpression inhibited virus infection (Yuan et al., 2019b). Six WRKY genes, *SolyWRKY41*, *SolyWRKY42*, *SolyWRKY53*, *SolyWRKY54*, *SolyWRKY80,* and *SolyWRKY81* obtained from tomato reduced TYLCV infection. Interaction analysis provided evidence of interaction between WRKY group III, isochorismate synthase (ICS), and Mitogen-Activated Protein Kinase5 (MAPK) in response to viruses (Huangfu et al., 2016).

### 4.4 Nematodes

Plant parasitic nematodes (PPNs) are economically important agricultural pests. Two classes exits i.e., cyst nematodes (CNs) and root knot nematodes (RKNs), causing together an estimated annual loss of US $80 billion. PPNs affect a wide range of hosts among economically important crops, i.e., *Solanum lycopersicum*, *Solanum tuberosum*, *Gossypium hirsutum*, *Glycine* max, *Oryza sativa*, *Zea mays,* and *Triticum aestivum* (Warmerdam et al., 2018). PPNs rich in ascaroside (Ascr# 18) induce the plant immune systems trigger production of jasmonic acid and salicylic acid, as well as trigger PTI and MAPKs. PPN’s induce secondary metabolite production in plants, i.e., chlorogenic acid, ethylene, and flavonoids in roots (Sato et al., 2019). These secondary metabolites reduced attraction of nematodes towards plant roots. Genes linked with synthesis of cytokinin, gibberellic acid, salicylic acid, jasmonates, and auxin signal responses are activated (Macharia et al., 2019). The role of TFs to reduce the negative impact of PPNs on plant growth is briefly summarized in the Table 2 and some examples are discussed below.

*SlWRKY75* is activated in *Solanum lycopersicum* by infection with *Meloidogyne javanica* and stimulate the JA pathway for regulation of the JA signaling mechanism. *CsWRKY23* is overexpressed in cucumber plants during infection of *Meloidogyne incognita* for contributing to early resistance (Macharia et al., 2019). *AtWRKY23* is overexpressed due to auxin stimulation at the feeding point of cyst nematode *Heterodera schachtii*. *OsWRKY11*, *OsWRKY70*, and *OsWRKY62* are upregulated in response to *Hirschmanniella oryza* attack. Similarly, *OsWRKY13*, *OsWRKY59*, and *OsWRKY62* are upregulated against RKN infection. Nineteen WRKY genes in *Solanum lycopersicum* responded to nematode infection, i.e., upregulation of *SlWRKY70* by salicylic acid, both *SlWRKY35* and *SlWRKY3* were activated (Chinnapandi et al., 2019). *AtWRKY33* was stimulated by JA and worked as a positive regulator against PPN attack. *AtWRKY33* overexpression along with different promoters conferred resistance against *Heterodera schachtii*. *SlWRKY45* is implicated in signal transduction pathways during accumulation of nematodes in the root zone (Chinnapandi et al., 2017). Five *Glycine* max WRKY genes i.e. *GmWRKY5*, *GmWRKY28*, *GmWRKY36*, *GmWRKY62*, and *GmWRKY154* were found more responsive against SCN and reduced 70% of its population, while *GmWRKY136*, *GmWRKY86*, *GmWRKY53*, and *GmWRKY52* showed moderate response by reducing 40–60% population and *GmWRKY71* and *GmWRKY8* showed a 10–30% control against SCN population (Yang et al., 2017a).

### 4.5 Pest attack

Changing climate is promoting the growth of herbivores and shortening their life cycles. However, rise in temperature is increasing chances of their appearance (Ximénez-Embún et al., 2017, Jamil et al., 2021; Ahmad et al., 2019). On the basis of feeding mode, insects are classified into different classes i.e. chewing insects such as beetles and caterpillars; consume plant tissues, whereas piercing and sucking insects feed on the vascular system for example aphids which insert their stylets into the phloem. Meanwhile, thrips combine sucking and rasping methods to feed on its host. Some mining type feeders such as larvae of certain beetles, moths, and flies form serpentine cavities when feeding between epidermal cells in leaf tissues and cause twisting or curling of leaves (Santamaria et al., 2018). Moreover, spider mites, a phytophagous acarian belonging to Tetranychus genus, pierce parenchyma cells and suck the contents (Bensoussan et al., 2016).

Some plants counter attack or activate emergency responses (Santamaria et al., 2013). The plant defense systems are activated when specific pattern recognition receptors (PRRs) detect phytophagous pests through herbivore-associated molecular patterns (HAMPs), microbe-associated molecular patterns (MAMPs), and damage associated molecular patterns (DAMPs). With recognition of molecular patterns, plants activates short-term downstream responses at the membrane levels, i.e., Ca^2+^ influx, potential depolarization, and generation of ROS or reactive nitrogen species (RNS) as a result, secretion of JA, SA, and ethylene starts, which activate TFs. TFs regulate expression of downstream-located genes through a cascade of CDPKs. These events, of recognition to response, take place within minutes to hours after herbivore attack (Santamaria et al., 2018).

Few TFs are upregulated in response to herbivore induced plant damage indicating their role in plant defense. *OsERF3* was upregulated in response to feeding of striped stem borer (SSB) in *Oryza sativa* and enhanced the transcript level of two MAPKs and two WRKY genes. As a result, the concentration of SA, JA, and trypsin protease inhibitor activity increased. *OsWRKY45* protected rice against infestation of Brown Plant hopper (*Nilaparvata lugens*) (Lu et al., 2011). *TaMYB19*, *TaMYB44,* and *TaMYB29* acted as co-regulators in phloem based defense response against English grain aphid in wheat. *AtMYB75* increased resistance against *Pieris brassicae* by modulating flavonoid metabolites. Overexpression of *GsMYB15* obtained from wild soybean increased *Arabidopsis* resistance against *Helicoverpa armigera* by a JA mediated insect response (Shen et al., 2018). *CmMYB19* increased resistance of *Chrysanthemum* against aphids by promoting a lignin biosynthesis pathway (An et al., 2019). *NbERF173* obtained from *Nicotiana benthamiana* provided resistance against *Phytophthora parasitica* (Yu et al., 2020). *TaWRKY53* induced resistance mechanisms against Russian wheat aphid in *Triticum aestivum* (Van Eck et al., 2014). Similarly, *OsWRKY53* provided resistance against *Chilo suppressalis* SSB (Hu et al., 2016).

## 5 Crop improvement techniques and TFs

**Fig. 3:**
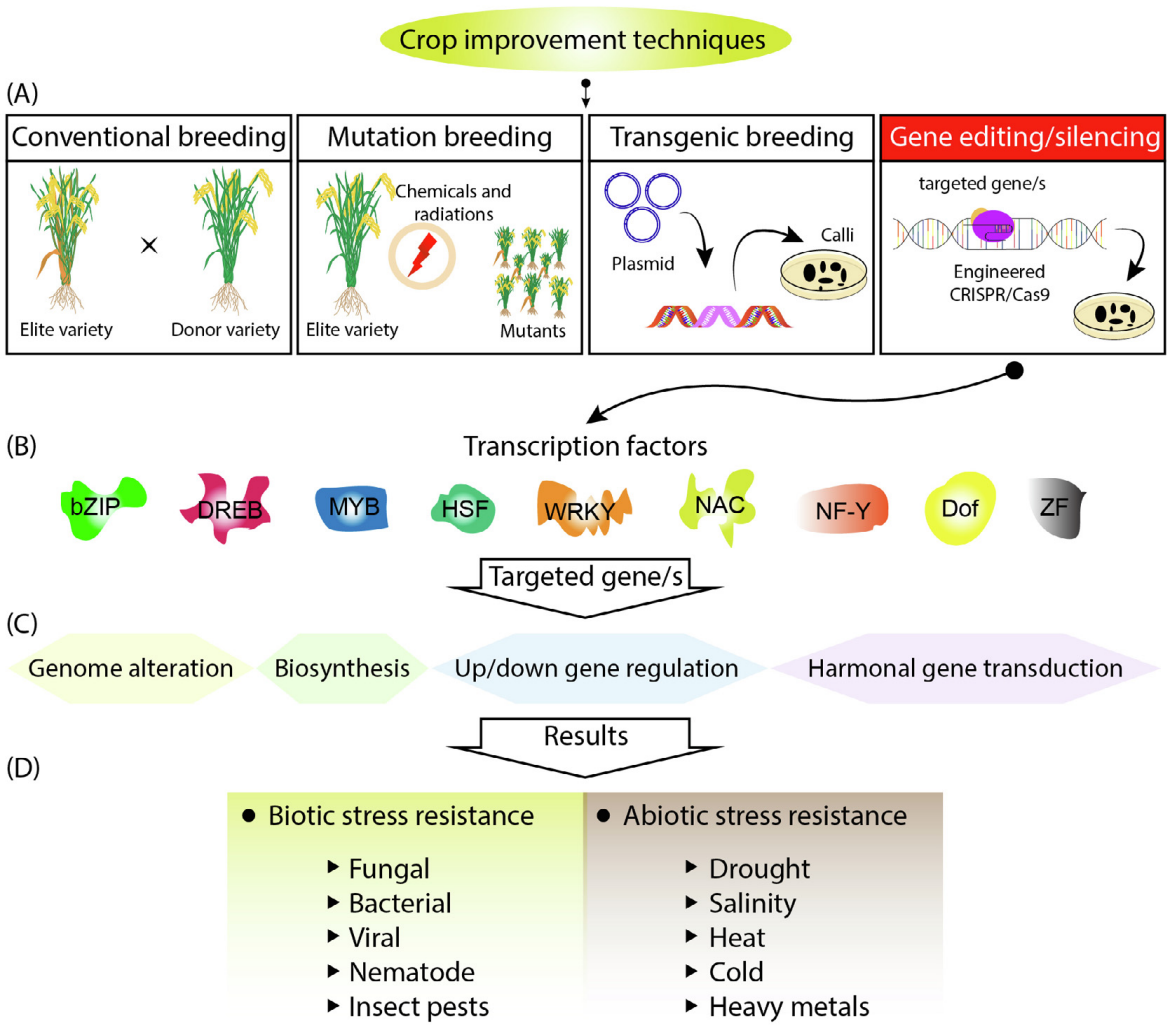
Schematic illustration of different crop improvement techniques particularly targeted modifications in TFs via gene editing/silencing for crop improvement. (**A**) Overview of different crop improvement techniques. (**B**) Different transcriptional factors that can be used for incorporation of biotic and abiotic stress tolerance in crops. (**C**) Different signal transduction pathways that are activated or modified by TFs. (**D**) Biotic and abiotic stresses that are alleviated by action of TFs.

The implication of TFs from signal perception to signal transduction and expression of stress responsive genes was summarized in this review. A single TF gene may respond to numerous stresses for example *SbNAC58* incorporate tolerance against multiple abiotic stresses i.e. drought, cold, and salinity stress (Baillo et al., 2019). TFs have potential to be exploited using different genome modifying molecular techniques for developing climate resilient crops (Table 3) as explained in Fig. 3.

**Table 3:** Crop improvement by targeting of TFs through gene silencing and transgenic and gene editing approaches.

Technique	Crop	Gene	Objective	Reference
RNAi silencing	*Arabidopsis thaliana*	*AtNAC042/JUB1, CBNAC/NTL9*	Bacterial Leaf spot	(Yuan et al., 2019a)
	*Triticum aestivum*	*TaNAC1, TaNAC21/22, TaNAC30*	Yellow rust and powdery mildew resistance	(Yuan et al., 2019a)
Genome editing	*Arabidopsis thaliana*	*AtWRKY11, AtWRKY70*	Pathogen resistance	(Ahmad et al., 2020)
	*Triticum aestivum*	*TaDREB2, TaERF3*	Drought tolerance	(Kim et al., 2018)
	*Oryza sativa*	*OsNAC2, OsNAC14, OsbZIP62*	Drought tolerance	(Yang et al., 2019)
		*OsERF922*	Rice blast tolerance	(Ahmad et al., 2020)
	*Brassica napus*	*BnWRKY11, BnWRKY70*	Pathogen resistance	(Ahmad et al., 2020)
Transgenic approach	*Arabidopsis thaliana*	*AtDREB1A, AtWRKY57*	Drought tolerance	(Rabara et al., 2014, Wu et al., 2019)
		*GmbZIP1, GmDREB2,*	Drought, cold, salinity tolerance	
		*ZmMYB3R, OsWRKY45*	Drought, salinity tolerance	
	*Triticum aestivum*	*AtDREB1A*	Drought tolerance	
		*GmbZIP1, GhDREB*	Drought, salinity, cold tolerance	
	*Oryza sativa*	*OsWRKY30, JERF1, OsbZIP23, OsbZIP46*	Drought tolerance	
		*SNAC1, SNAC2*	Drought, salinity tolerance	
	*Nicotiana tabacum*	*AtDREB1A, GmERF3*	Drought tolerance

Gene silencing through RNAi provides a platform for exploring the role of different TFs in plant development and in response to various stresses. RNAi uses double-stranded RNA to activate ribonucleases to target homologous mRNA and degrade it. The resulting phenotypes are either null or partially affected. Thus RNAi can help to elucidate role of different TFs under biotic and abiotic stresses (Agrawal et al., 2003). This knowledge can be exploited by incorporating favorable alleles in suitable genetic backgrounds, and using different biotechnological tools, for stress tolerance. RNAi silencing could be used to knockdown TFs, which promote disease development. It can also be useful for knocking out undesirable TFs, which promote the development of stresses. *AtNAC042/JUB1*, *CBNAC/NTL9* promotes bacterial leaf spot of *Arabidopsis.* Similarly, *TaNAC1*, *TaNAC21/22*, *TaNAC30* promotes yellow rust and downy mildew attack on wheat. Knocking down these TFs in various experiments showed progress and have slowed down the disease establishment processes (Yuan et al., 2019b).

Different genome editing tools, such as ZF-nucleases (ZFNs), homing endonucleases or mega nucleases, and transcription activator-like effector nucleases (TALENs) create targeted double-strand breaks that promote recombination at a specific locus and have potential in exploring the role of different TFs (Rabara et al., 2014). Similarly, Clustered Regulatory Interspaced Short Palindromic Repeats (CRISPR) are used to establish knockout lines of TF genes for functional genetics. In the CRISPR/Cas9 system, the genomic target site is cleaved by Cas9, located at the site by the guide RNA (gRNA) with which it complexes. As a result, a double stranded break occurs at the target site, the repair of which causes mutations in the form of insertions or deletions or in some cases frameshifts. These mutants can clarify the role of the TF under consideration (Ahmad et al., 2020; Monsur et al., 2020).

Marker assisted breeding (MAB) has a wide variety of applications (Jamil et al., 2020b; Jamil et al., 2020c) in stacking of multiple genes in crop plants for various purposes and had been widely used in studying wheat rust (Liu et al., 2020). All we need to find out for marker-assisted breeding is the tight linkage of a molecular marker (with TF as our interest). MAB was used previously for *MdMYB1*, associated with apple fruit skin color. *MdMYB1* imparts red color to the fruit whereas its absence results in green color. A dCAPS marker was developed for selection of fruit color in apples at early plant developmental stages using MAB (Zhu et al., 2011). Another very successful example of MAB is the introduction of the *SUB1* region into rice genetic backgrounds, which increases submergence tolerance without effecting yield, grain quality or development (Oladosu et al., 2020). Similar marker systems could be developed for WRKY and other TFs responding to various biotic and abiotic stresses for foreground selection prior to plantation. This will save time and labor, and will facilitate stacking of TF genes for multiple responsiveness in crop plants.

Development of transgenic plants using TFs has a wide variety of potential applications in development of stress tolerant crops. An example is the production of drought tolerant tobacco plants through the use of *MdDREB76* from apple (Sharma et al., 2019), or the use of wheat *TaNAC29* to enhance salt and drought tolerance in *Arabidopsis* (Huang et al., 2015), and many more examples exists in the literature (Table 3). During development of transgenic plants, TFs is most frequently put under the control of a constitutive promotor, i.e., *CAMV35S* that is expressed in each cell at all growth stages of the plants. Thus, great potential exists for development of transgenic plants using multiple stresses responsive TFs and evaluating their expression in various crops through development of transgenic crops. However, in successive generations of transgenic, rigorous selection criteria should be used to select stress tolerant plants with no negative effects (Fahad et al., 2017).

## 6 Ductility and flexibility in TFs to carry out their function

TFs are the most flexible proteins in nature, and this characteristic is very important to conduct regulatory function. It is predicted that 83–94% of TFs possess extended regions of ductile/disordered residues in eukaryotic organisms. It is observed that organismic complexity is positively and strongly correlated with total number of TFs, the number of their spliced variants, and their total disordered residue content. Transcriptional factor families that take part in cell cycle, cell size, cell proliferation, and cell differentiation have more disordered residues and are more flexible. These evidence suggested that increasing TFs are an important factor for increasing organismic complexity (Yruela et al., 2017). This ductility of TFs helps plants combat multiple abiotic stress responses by acting as protein chaperones or protecting other cellular components and structures. TFs have complex and versatile networks to efficiently respond to environmental changes. TF disorder plays an important role in plants, providing them with a fast mechanism to obtain complex, interconnected, and versatile molecular networks (Yruela, 2015).

## 7 Future perspectives

TFs have great potential for boosting the yield and stress tolerance in field crops. Though, significant achievements have been made in unraveling the potential role of TFs under various biotic and abiotic stresses (Li et al., 2020c). But, pathways explaining the role of TFs under stresses are yet to be explored. Approximately 50 TFs families have been reported and among those, less than ten including WRKY, DREB, NAC, HSF, MYB, ZF-s, Dof, bZIP, and NAC have been functionally characterized under various biotic and abiotic stresses in different crops. Meanwhile, there is an immense potential to explore the role of remaining TF families in plants’ health and yield improvement, and utilize them in crop improvement programs according to their role. Moreover, the information may open new horizons for young researchers to contribute in crop improvement by utilizing different TFs. Similarly, there is an opportunity to establish crosstalk between different TF gene families and then find how they respond under stress conditions Focus should shift towards development of climate resilient crops with biotic as well as abiotic stress tolerance. For instance, majority of WRKY TF genes have their positive role against biotic and abiotic stresses in plants (Li et al., 2020c). Nevertheless, other TFs also resist both biotic as well as abiotic stresses simultaneously. There is a need to identify TF genes that interplay during different stresses. TF genes expressing under multiple stresses should be privileged for breeding climate-smart varieties through conventional as well as modern plant breeding tools.

Transgenic crops promises to be a good source of resistance against biotic and abiotic stresses. However, delivery of TFs to various genetic backgrounds using transgenic technologies still pose a great challenge to researchers due to unexplored metabolic pathways. Nevertheless, the role of many TFs has been fully characterized (Table 1, Table 2) and those can be utilized in breeding climate-smart crops. Currently, there are many new and smart breeding techniques such as genome editing, speed breeding etc., that can be used for developing climate resilient crops by using certain appropriate TFs. For example, *AtMYB14* and *AtMYB15* TFs play their negative role against abiotic stress tolerance in *Arabidopsis* (Table 1). This means that these TFs are involved in the activation of such gene that promotes sensitivity against stresses in plants. Thus, if we manipulate these TFs or their binding sites in the promoter of their respective gene/s then they will be unable to bind and regulate their respective sensitivity gene/s. Ultimately, plants will be more tolerant to the corresponding stress because the TFs and/or gene that was facilitating the proliferation of biotic and/or abiotic stress has been knocked out. Likewise, similar proteins in other species can also be found through certain bioinformatics analysis and can be targeted through genome editing tools for manipulating their negative role in plants.

Concomitantly, many negative regulators can also be found against biotic stress tolerance. For instance, some genes of NAC TFs i.e., *TaNAC1* promotes yellow rust and downy mildew in wheat thus effect wheat growth and yield. On the other hand, it can be seens from tables (Tables 1 and 2) that maximum of the TFs has their positive role in tolerance against biotic and abiotic stresses in plants, hence over-expression of these TFs can improve tolerance against biotic and abiotic stresses in different plant species. Although, these techniques are robust, efficient and have been widely used in plants since last decade, but due to certain limitations and issues such as “off-target effects”, these techniques need to be improved and might not be enough to achieve global food security with current pace of development (Deniaud et al., 2009). Recently, many efforts have been made to resolve the issue of “Off-target effects” and make the genome editing tool as a model and robust tool for genome modifications. For example, use of tissue specific promotors (*SynR1* and *SynR2* are root specific), as explained by Ali et al. (Ali and Kim, 2019), would help in overcoming the off-target affects.

Moreover, most of the functional studies, involved in exploring the role of TFs, are conducted in model plants i.e., tobacco (Sharma et al., 2019) and *Arabidopsis* (Huang et al., 2015), which are relatively easy to handle. The focus should be shifted to cultivated crops (i.e., wheat, rice, maize, and other field crops) for biotic and abiotic stress tolerance. Although, reports are available on the transformation of TFs in field crops, but stacking of multiple stress responsive TFs is just a beginning. Similarly, the role of TFs against heavy metal stress, nematodes and insect attacks needs more attention as these are neglected fields and limited studies were conducted in these directions. Another area of improvement is development of functional marker systems, i.e., SSR markers, SNPs, or dCAPS for MAB of different TFs. To the best of our knowledge, only few examples exist in literature related to the development of functional marker systems for characterization of TFs (Zhu et al., 2011, Liu et al., 2020, Oladosu et al., 2020). Functional markers will help identification of TFs in successive generations for marker-assisted crop improvement. Taken together, TFs have immense potential and opportunities for crop improvement and to achieve global food security.

## 8 Conclusions

Genome wide studies of different plant TFs gene families have played crucial role in unravelling the role of TFs in various metabolic pathways and identify the key genes which respond to biotic and abiotic stresses. These studies have provided insights about the potent role of TFs in combating different environmental stresses and their utilization to obtain relatively high yield under stress conditions. Different biotic stresses (i.e., bacterial, fungal and viral diseases, insects and nematodes) and abiotic stresses (i.e., drought, waterlogging, heat, cold, salinity, and heavy metals) are becoming an alarming threat to crop productivity due to changing climate. There is an urgent need for the development of biotic and abiotic stress tolerant crops by targeting different genes and/or their regulators. In this regard, different crop improvement approaches including RNAi silencing, genome editing, speed breeding etc., promise to deliver safer food to human beings and ensure food security. TFs should be exploited by new breeding tools for developing climate-resilient varieties. These varieties will not only combat different biotic and abiotic factors but also improve yield and overcome food insecurity.

Funding

The publication of present work was supported by the National Key Research and Development Program of China (grant no. 2017YFC0504704), and the National Natural Science Foundation of China (51669034, 41761068, 51809224).

## CRediT authorship contribution statement

**Rahil Shahzad**: Conceptualization, Data curation, Software, Writing - original draft, Writing - review & editing. **Shakra Jamil**: Conceptualization, Data curation, Supervision, Writing - original draft, Writing - review & editing. **Shakeel Ahmad**: Data curation, Software, Writing - original draft, Writing - review & editing. **Amina Nisar**: Data curation, Software, Writing - original draft. **Zarmaha Amina**: Data curation, Software, Writing - original draft. **Shazmina Saleem**: Data curation, Writing - original draft. **Muhammad Zaffar Iqbal**: Funding acquisition, Supervision, Writing - review & editing. **Rana Muhammad Atifc**: Data curation, Supervision, Writing - review & editing. **Xiukang Wang**: Data curation, Funding acquisition, Writing - review & editing.

## Declaration of Competing Interest

The authors declare that they have no known competing financial interests or personal relationships that could have appeared to influence the work reported in this paper.

## References

[b0005] Agrawal N., Dasaradhi P., Mohmmed A., Malhotra P., Bhatnagar R.K., Mukherjee S.K. (2003). RNA interference: biology, mechanism, and applications. Microbiol. Mol. Biol. Rev..

[bib838] Ahmad S., Cheema H.M.N., Khan A.A., Khan R.S.A., Ahmad J.N. (2019). Resistance status of *Helicoverpa armigera* against *Bt* cotton in Pakistan. Transgenic Res..

[b0010] Ahmad S., Wei X., Sheng Z., Hu P., Tang S. (2020). CRISPR/Cas9 for development of disease resistance in plants: recent progress, limitations and future prospects. Briefings Funct. Genomics.

[b0015] Åkerfelt M., Morimoto R.I., Sistonen L. (2010). Heat shock factors: integrators of cell stress, development and lifespan. Nat. Rev. Mol. Cell Biol..

[b0020] Ali S., Kim W.-C. (2019). A fruitful decade using synthetic promoters in the improvement of transgenic plants. Front. Plant Sci..

[b0025] Ali U., Zhong M., Shar T., Fiaz S., Xie L., Jiao G., Ahmad S., Sheng Z., Tang S., Wei X. (2019). The Influence of pH on Cadmium Accumulation in Seedlings of Rice (Oryza sativa L.). J. Plant Growth Regul..

[b0030] Allu A.D., Brotman Y., Xue G.P., Balazadeh S. (2016). Transcription factor ANAC032 modulates JA/SA signalling in response to Pseudomonas syringae infection. EMBO Rep..

[b0035] Amirbakhtiar N., Ismaili A., Ghaffari M.R., Firouzabadi F.N., Shobbar Z.-S. (2019). Transcriptome response of roots to salt stress in a salinity-tolerant bread wheat cultivar. PLoS ONE.

[b0040] Amorim, A., Lidiane, L., Da Fonseca Dos Santos, R., Pacifico Bezerra Neto, J., Guida-Santos, M., Crovella, S., and Maria Benko-Iseppon, A., 2017. Transcription factors involved in plant resistance to pathogens. Current Protein and Peptide Science 18, 335-351. 10.2174/1389203717666160619185308 27323805

[b0045] An C., Sheng L., Du X., Wang Y., Zhang Y., Song A., Jiang J., Guan Z., Fang W., Chen F. (2019). Overexpression of CmMYB15 provides chrysanthemum resistance to aphids by regulating the biosynthesis of lignin. Hortic. Res..

[b0050] Ashrafijou M., Noori S.S., Darbandi A.I., Saghafi S. (2010). Effect of salinity and radiation on proline accumulation in seeds of canola (Brassica napus L.). Plant Soil Environ..

[b0055] Baillo E.H., Kimotho R.N., Zhang Z., Xu P. (2019). Transcription factors associated with abiotic and biotic stress tolerance and their potential for crops improvement. Genes.

[b0060] Banerjee A., Roychoudhury A. (2017). Abscisic-acid-dependent basic leucine zipper (bZIP) transcription factors in plant abiotic stress. Protoplasma.

[b0065] Barickman T.C., Simpson C.R., Sams C.E. (2019). Waterlogging causes early modification in the physiological performance, carotenoids, chlorophylls, proline, and soluble sugars of cucumber plants. Plants.

[b0070] Bensoussan N., Santamaria M.E., Zhurov V., Diaz I., Grbić M., Grbić V. (2016). Plant-herbivore interaction: dissection of the cellular pattern of Tetranychus urticae feeding on the host plant. Front. Plant Sci..

[b0075] Borrego-Benjumea A., Carter A., Tucker J.R., Yao Z., Xu W., Badea A. (2020). Genome-Wide Analysis of Gene Expression Provides New Insights into Waterlogging Responses in Barley (Hordeum vulgare L.). Plants.

[b0080] Buttimer C., Mcauliffe O., Ross R.P., Hill C., O’mahony J., Coffey A. (2017). Bacteriophages and bacterial plant diseases. Front. Microbiol..

[b0085] Calil I.P., Fontes E.P. (2017). Plant immunity against viruses: antiviral immune receptors in focus. Ann. Bot..

[b0090] Casaretto J.A., El-Kereamy A., Zeng B., Stiegelmeyer S.M., Chen X., Bi Y.-M., Rothstein S.J. (2016). Expression of OsMYB55 in maize activates stress-responsive genes and enhances heat and drought tolerance. BMC Genomics.

[b0095] Cerda R., Avelino J., Gary C., Tixier P., Lechevallier E., Allinne C. (2017). Primary and secondary yield losses caused by pests and diseases: Assessment and modeling in coffee. PLoS ONE.

[b0100] Chaffei C. (2003). Nitrogen metabolism of tomato under cadmium stress conditions. J. Plant Nutr..

[b0105] Chen F., Hu Y., Vannozzi A., Wu K., Cai H., Qin Y., Mullis A., Lin Z., Zhang L. (2017). The WRKY transcription factor family in model plants and crops. Crit. Rev. Plant Sci..

[b0110] Chen H., Liu L., Wang L., Wang S., Cheng X. (2016). VrDREB2A, a DREB-binding transcription factor from Vigna radiata, increased drought and high-salt tolerance in transgenic Arabidopsis thaliana. J. Plant Res..

[b0115] Chinnapandi B., Bucki P., Braun Miyara S. (2017). SlWRKY45, nematode-responsive tomato WRKY gene, enhances susceptibility to the root knot nematode; M. javanica infection. Plant Signaling Behav..

[b0120] Chinnapandi B., Bucki P., Fitoussi N., Kolomiets M., Borrego E., Braun Miyara S. (2019). Tomato SlWRKY3 acts as a positive regulator for resistance against the root-knot nematode Meloidogyne javanica by activating lipids and hormone-mediated defense-signaling pathways. Plant Signaling Behav..

[b0125] Ciarmiello, L.F., Woodrow, P., Piccirillo, P., De Luca, A., and Carillo, P., 2014. “Transcription factors and environmental stresses in plants,” in Emerging Technologies and Management of Crop Stress Tolerance. Elsevier), 57-78.

[b0130] Colmer T.D., Greenway H. (2011). Ion transport in seminal and adventitious roots of cereals during O2 deficiency. J. Exp. Bot..

[b0135] Crafts-Brandner S.J., Salvucci M.E. (2002). Sensitivity of photosynthesis in a C4 plant, maize, to heat stress. Plant Physiol..

[b0140] Deniaud E., Baguet J., Chalard R., Blanquier B., Brinza L., Meunier J., Michallet M.-C., Laugraud A., Ah-Soon C., Wierinckx A. (2009). Overexpression of transcription factor Sp1 leads to gene expression perturbations and cell cycle inhibition. PLoS ONE.

[b0145] Dennis E.S., Dolferus R., Ellis M., Rahman M., Wu Y., Hoeren F., Grover A., Ismond K., Good A., Peacock W. (2000). Molecular strategies for improving waterlogging tolerance in plants. J. Exp. Bot..

[b0150] Du C., Zhao P., Zhang H., Li N., Zheng L., Wang Y. (2017). The Reaumuria trigyna transcription factor RtWRKY1 confers tolerance to salt stress in transgenic Arabidopsis. J. Plant Physiol..

[b0155] Dubos C., Stracke R., Grotewold E., Weisshaar B., Martin C., Lepiniec L. (2010). MYB transcription factors in Arabidopsis. Trends Plant Sci..

[b0160] Erpen L., Devi H.S., Grosser J.W., Dutt M. (2018). Potential use of the DREB/ERF, MYB, NAC and WRKY transcription factors to improve abiotic and biotic stress in transgenic plants. Plant Cell. Tissue Organ Culture (PCTOC).

[b0165] Eulgem T., Rushton P.J., Robatzek S., Somssich I.E. (2000). The WRKY superfamily of plant transcription factors. Trends Plant Sci..

[b0170] Fahad S., Bajwa A.A., Nazir U., Anjum S.A., Farooq A., Zohaib A., Sadia S., Nasim W., Adkins S., Saud S. (2017). Crop production under drought and heat stress: plant responses and management options. Front. Plant Sci..

[b0175] Feng Y., Yao Z., Klionsky D.J. (2015). How to control self-digestion: transcriptional, post-transcriptional, and post-translational regulation of autophagy. Trends Cell Biol..

[b0180] Figueiredo D.D., Barros P.M., Cordeiro A.M., Serra T.S., Lourenço T., Chander S., Oliveira M.M., Saibo N.J. (2012). Seven zinc-finger transcription factors are novel regulators of the stress responsive gene OsDREB1B. J. Exp. Bot..

[b0185] Finatto T., Viana V.E., Woyann L.G., Busanello C., Maia L.C.D., Oliveira A.C.D. (2018). Can WRKY transcription factors help plants to overcome environmental challenges?. Genet. Mol. Biol..

[b0190] Fujita Y., Fujita M., Satoh R., Maruyama K., Parvez M.M., Seki M., Hiratsu K., Ohme-Takagi M., Shinozaki K., Yamaguchi-Shinozaki K. (2005). AREB1 is a transcription activator of novel ABRE-dependent ABA signaling that enhances drought stress tolerance in Arabidopsis. Plant Cell.

[b0195] Fukao T., Barrera-Figueroa B.E., Juntawong P., Peña-Castro J.M. (2019). Submergence and Waterlogging Stress in Plants: A Review Highlighting Research Opportunities and Understudied Aspects. Front. Plant Sci..

[b0200] Golldack D., Lüking I., Yang O. (2011). Plant tolerance to drought and salinity: stress regulating transcription factors and their functional significance in the cellular transcriptional network. Plant Cell Rep..

[b0205] Guo M., Liu J.-H., Ma X., Luo D.-X., Gong Z.-H., Lu M.-H. (2016). The plant heat stress transcription factors (HSFs): structure, regulation, and function in response to abiotic stresses. Front. Plant Sci..

[b0210] Hamamouch N., Winkel B.S., Li C., Davis E.L. (2020). Modulation of Arabidopsis Flavonol Biosynthesis Genes by Cyst and Root-Knot Nematodes. Plants.

[b0215] Hasegawa P.M. (2013). Sodium (Na+) homeostasis and salt tolerance of plants. Environ. Exp. Bot..

[b0220] He X.J., Mu R.L., Cao W.H., Zhang Z.G., Zhang J.S., Chen S.Y. (2005). AtNAC2, a transcription factor downstream of ethylene and auxin signaling pathways, is involved in salt stress response and lateral root development. Plant J..

[b0225] Hinojosa L., Matanguihan J.B., Murphy K.M. (2019). Effect of high temperature on pollen morphology, plant growth and seed yield in quinoa (Chenopodium quinoa Willd.). J. Agron. Crop Sci..

[b0230] Hofmann N.R. (2013). A NAC Transcription Factor for Flooding: SHYG Helps Plants Keep Their Leaves in the Air. Am Soc Plant Biol.

[b0235] Hong C., Cheng D., Zhang G., Zhu D., Chen Y., Tan M. (2017). The role of ZmWRKY4 in regulating maize antioxidant defense under cadmium stress. Biochem. Biophys. Res. Commun..

[b0240] Hou H., Zhao L., Zheng X., Gautam M., Yue M., Hou J., Chen Z., Wang P., Li L. (2019). Dynamic changes in histone modification are associated with upregulation of Hsf and rRNA genes during heat stress in maize seedlings. Protoplasma.

[b0245] Hu L., Ye M., Li R., Lou Y. (2016). OsWRKY53, a versatile switch in regulating herbivore-induced defense responses in rice. Plant Signaling Behav..

[b0250] Huang Q., Wang Y., Li B., Chang J., Chen M., Li K., Yang G., He G. (2015). TaNAC29, a NAC transcription factor from wheat, enhances salt and drought tolerance in transgenic Arabidopsis. BMC Plant Biol..

[b0255] Huang Y., Li M.-Y., Wu P., Xu Z.-S., Que F., Wang F., Xiong A.-S. (2016). Members of WRKY Group III transcription factors are important in TYLCV defense signaling pathway in tomato (Solanum lycopersicum). BMC Genomics.

[b0260] Huang Y., Li T., Xu Z.-S., Wang F., Xiong A.-S. (2017). Six NAC transcription factors involved in response to TYLCV infection in resistant and susceptible tomato cultivars. Plant Physiol. Biochem..

[b0265] Huangfu J., Li J., Li R., Ye M., Kuai P., Zhang T., Lou Y. (2016). The transcription factor OsWRKY45 negatively modulates the resistance of rice to the brown planthopper Nilaparvata lugens. Int. J. Mol. Sci..

[b0270] Hussain H.A., Hussain S., Khaliq A., Ashraf U., Anjum S.A., Men S., Wang L. (2018). Chilling and drought stresses in crop plants: implications, cross talk, and potential management opportunities. Front. Plant Sci..

[b0275] Hussain M.I., Reigosa M.J. (2015). Characterization of xanthophyll pigments, photosynthetic performance, photon energy dissipation, reactive oxygen species generation and carbon isotope discrimination during artemisinin-induced stress in *Arabidopsis thaliana*. PLoS ONE.

[b0280] Iuchi S. (2001). Three classes of C2H2 zinc finger proteins. Cell Mol. Life Sci. CMLS.

[b0285] Jalmi S.K., Bhagat P.K., Verma D., Noryang S., Tayyeba S., Singh K., Sharma D., Sinha A.K. (2018). Traversing the links between heavy metal stress and plant signaling. Front. Plant Sci..

[b0290] Jamil S., Shahzad R., Ahmad S., Fatima R., Zahid R., Anwar M., Iqbal M.Z., Wang X. (2020). Role of Genetics, Genomics and Breeding approaches to combat stripe rust of wheat. Front. Nutrit..

[bib839] Jamil S., Shahzad R., Kanwal S., Yasmeen E., Rahman S.U., Iqbal M.Z. (2020). DNA Fingerprinting and Population Structure of Date Palm Varieties Grown in Punjab Pakistan using Simple Sequence Repeat Markers. Int. J. Agri. Bio..

[bib840] Jamil S., Shahzad` R., Yasmeen E., Rahman S.U., Younas M., Iqbal M.Z. (2020). DNA fingerprinting of pakistani maize hybrids and parental lines using simple sequence repeat markers.. Pak. J. Bot..

[b0300] Jamir I., Mandal A.K., Devi A.P., Bhattacharjee T., Maurya P.K., Dutta S., Chattopadhyay A., Pramanik K., Banik S. (2020). Screening of genotypes against viral diseases and assessment of yield loss due to yellow vein mosaic virus in okra grown in the eastern part of India. Indian Phytopathol..

[b0295] Jamil S., Shahzad R., Rahman S.U., Iqbal M.Z., Yaseen M., Ahmad S., Fatima R. (2021). The level of Cry1Ac endotoxin and its efficacy against H. armigera in Bt cotton at large scale in Pakistan. GM Crops Food.

[b0305] Javed T., Shabbir R., Ali A., Afzal I., Zaheer U., Gao S.-J. (2020). Transcription Factors in Plant Stress Responses: Challenges and Potential for Sugarcane Improvement. Plants.

[b0310] Jiang J., Ma S., Ye N., Jiang M., Cao J., Zhang J. (2017). WRKY transcription factors in plant responses to stresses. J. Integr. Plant Biol..

[b0315] Jin Q., Xu Y., Mattson N., Li X., Wang B., Zhang X., Jiang H., Liu X., Wang Y., Yao D. (2017). Identification of submergence-responsive microRNAs and their targets reveals complex miRNA-mediated regulatory networks in lotus (Nelumbo nucifera Gaertn). Front. Plant Sci..

[b0320] Joshi R., Wani S.H., Singh B., Bohra A., Dar Z.A., Lone A.A., Pareek A., Singla-Pareek S.L. (2016). Transcription factors and plants response to drought stress: current understanding and future directions. Front. Plant Sci..

[b0325] Jung K.-H., Ko H.-J., Nguyen M.X., Kim S.-R., Ronald P., An G. (2012). Genome-wide identification and analysis of early heat stress responsive genes in rice. J. Plant Biol..

[b0330] Kaur G., Subramanian S. (2016). Classification of the treble clef zinc finger: noteworthy lessons for structure and function evolution. Sci. Rep..

[b0335] Kaymakanova M. (2009). Effect of salinity on germination and seed physiology in bean (Phaseolus vulgaris L.). Biotechnol. Biotechnol. Equip..

[b0340] Kidokoro, S., Watanabe, K., Ohori, T., Moriwaki, T., Maruyama, K., Mizoi, J., Myint Phyu Sin Htwe, N., Fujita, Y., Sekita, S., and Shinozaki, K., 2015. Soybean DREB 1/CBF‐type transcription factors function in heat and drought as well as cold stress‐responsive gene expression. The Plant Journal 81, 505-518. 10.1111/tpj.12746 25495120

[b0345] Kim D., Alptekin B., Budak H. (2018). CRISPR/Cas9 genome editing in wheat. Funct. Integr. Genomics.

[b0350] Kim H.J., Nam H.G., Lim P.O. (2016). Regulatory network of NAC transcription factors in leaf senescence. Curr. Opin. Plant Biol..

[b0355] Kimotho R.N., Baillo E.H., Zhang Z. (2019). Transcription factors involved in abiotic stress responses in Maize (Zea mays L.) and their roles in enhanced productivity in the post genomics era. PeerJ.

[b0360] Kudo M., Kidokoro S., Yoshida T., Mizoi J., Todaka D., Fernie A.R., Shinozaki K., Yamaguchi-Shinozaki K. (2017). Double overexpression of DREB and PIF transcription factors improves drought stress tolerance and cell elongation in transgenic plants. Plant Biotechnol. J..

[b0365] Kumar A., Jaiswal J.P., Sharma N., Gupta S., Kumar A. (2018). Understanding the molecular basis of differential grain protein accumulation in wheat (Triticum aestivum L.) through expression profiling of transcription factors related to seed nutrients storage. 3. Biotech.

[b0370] Lan Thi Hoang, X., Du Nhi, N.H., Binh Anh Thu, N., Phuong Thao, N., and Phan Tran, L.-S., 2017. Transcription factors and their roles in signal transduction in plants under abiotic stresses. Curr. Genomics 18, 483-497. 10.2174/1389202918666170227150057 PMC5684650 29204078

[b0375] Lee H., Fischer R.L., Goldberg R.B., Harada J.J. (2003). Arabidopsis LEAFY COTYLEDON1 represents a functionally specialized subunit of the CCAAT binding transcription factor. Proc. Natl. Acad. Sci..

[b0380] Leng P., Zhao J. (2019). Transcription factors as molecular switches to regulate drought adaptation in maize. Theor. Appl. Genet..

[b0385] Li B., Fan R., Yang Q., Hu C., Sheng O., Deng G., Dong T., Li C., Peng X., Bi F. (2020). Genome-Wide Identification and Characterization of the NAC Transcription Factor Family in Musa Acuminata and Expression Analysis during Fruit Ripening. Int. J. Mol. Sci..

[b0390] Li D., Fu F., Zhang H., Song F. (2015). Genome-wide systematic characterization of the bZIP transcriptional factor family in tomato (Solanum lycopersicum L.). BMC Genomics.

[b0395] Li D., Liu P., Yu J., Wang L., Dossa K., Zhang Y., Zhou R., Wei X., Zhang X. (2017). Genome-wide analysis of WRKY gene family in the sesame genome and identification of the WRKY genes involved in responses to abiotic stresses. BMC Plant Biol..

[b0400] Li J., Gao K., Khan W.U., Yang X., Yang X., Zhao T., Chen Z., An X. (2020). Genome-wide analysis of the poplar NF-Y gene family and its expression in floral bud development of Populus tomentosa. Trees.

[b0405] Li L., Zheng W., Zhu Y., Ye H., Tang B., Arendsee Z.W., Jones D., Li R., Ortiz D., Zhao X. (2015). QQS orphan gene regulates carbon and nitrogen partitioning across species via NF-YC interactions. Proc. Natl. Acad. Sci..

[b0410] Li S., Li K., Ju Z., Cao D., Fu D., Zhu H., Zhu B., Luo Y. (2016). Genome-wide analysis of tomato NF-Y factors and their role in fruit ripening. BMC Genomics.

[b0415] Li S., Zhou X., Chen L., Huang W., Yu D. (2010). Functional characterization of Arabidopsis thaliana WRKY39 in heat stress. Mol. Cells.

[b0420] Li W., Pang S., Lu Z., Jin B. (2020). Function and Mechanism of WRKY Transcription Factors in Abiotic Stress Responses of Plants. Plants.

[b0425] Li X., Guo C., Gu J., Duan W., Zhao M., Ma C., Du X., Lu W., Xiao K. (2014). RETRACTED: Overexpression of VP, a vacuolar H+-pyrophosphatase gene in wheat (Triticum aestivum L.), improves tobacco plant growth under Pi and N deprivation, high salinity, and drought. J. Exp. Bot..

[b0430] Liao Y., Zou H.-F., Wei W., Hao Y.-J., Tian A.-G., Huang J., Liu Y.-F., Zhang J.-S., Chen S.-Y. (2008). Soybean GmbZIP44, GmbZIP62 and GmbZIP78 genes function as negative regulator of ABA signaling and confer salt and freezing tolerance in transgenic Arabidopsis. Planta.

[b0435] Ling H., Huang N., Wu Q., Su Y., Peng Q., Ahmed W., Gao S., Su W., Que Y., Xu L. (2018). Transcriptional insights into the sugarcane-sorghum mosaic virus interaction. Tropical Plant Biol..

[b0440] Liu M., Ma Z., Sun W., Huang L., Wu Q., Tang Z., Bu T., Li C., Chen H. (2019). Genome-wide analysis of the NAC transcription factor family in Tartary buckwheat (*Fagopyrum tataricum*). BMC Genomics.

[b0445] Liu R., Lu J., Zhou M., Zheng S., Liu Z., Zhang C., Du M., Wang M., Li Y., Wu Y. (2020). Developing stripe rust resistant wheat (Triticum aestivum L.) lines with gene pyramiding strategy and marker-assisted selection. Genet. Resour. Crop Evol..

[b0450] Lohani N., Golicz A.A., Singh M.B., Bhalla P.L. (2019). Genome-wide analysis of the Hsf gene family in Brassica oleracea and a comparative analysis of the Hsf gene family in B. oleracea, B. rapa and B. napus. Funct. Integr. Genomics.

[b0455] Lu J., Ju H., Zhou G., Zhu C., Erb M., Wang X., Wang P., Lou Y. (2011). An EAR-motif-containing ERF transcription factor affects herbivore-induced signaling, defense and resistance in rice. Plant J..

[b0460] Luo J., Xia W., Cao P., Xiao Z.A., Zhang Y., Liu M., Zhan C., Wang N. (2019). Integrated transcriptome analysis reveals plant hormones jasmonic acid and salicylic acid coordinate growth and defense responses upon fungal infection in poplar. Biomolecules.

[b0465] Macharia T.N., Bellieny-Rabelo D., Moleleki L.N. (2019). Transcriptional profiling of potato (Solanum tuberosum L.) during a compatible interaction with the root-knot nematode, Meloidogyne javanica. BioRxiv.

[b0470] Maleva M., Nekrasova G., Borisova G., Chukina N., Ushakova O. (2012). Effect of heavy metals on photosynthetic apparatus and antioxidant status of Elodea. Russ. J. Plant Physiol..

[b9000] Mansfield J., Genin S., Magori S., Citovsky V., Sriariyanum M., Ronald P., Dow M.A.X., Verdier V., Beer S.V., Machado M.A., Toth I.A.N. (2012). Top 10 plant pathogenic bacteria in molecular plant pathology. Mol. Plant Pathol.

[b0475] Mall R., Gupta A., Sonkar G. (2017). Effect of climate change on agricultural crops,“ in Current developments in biotechnology and bioengineering. Elsevier.

[b0480] Matthews R. (2012). Plant virology.

[bib837] Monsur M.B., Shao G., Lv Y., Ahmad S., Wei X., Hu P., Tang S. (2020). Base Editing: The Ever Expanding Clustered Regularly Interspaced Short Palindromic Repeats (CRISPR) Tool Kit for Precise Genome Editing in Plants. Genes.

[b0485] Moon S.-J., Min M.K., Kim J., Kim D.Y., Yoon I.S., Kwon T.R., Byun M.O., Kim B.-G. (2019). Ectopic expression of OsDREB1G, a member of the OsDREB1 subfamily, confers cold stress tolerance in rice. Front. Plant Sci..

[b0490] Muthiah M., Ramadass A., Amalraj R.S., Palaniyandi M., Rasappa V. (2013). Expression profiling of transcription factors (TFs) in sugarcane X Colletotrichum falcatum interaction. J. Plant Biochem. Biotechnol..

[b0495] Nardini M., Gnesutta N., Donati G., Gatta R., Forni C., Fossati A., Vonrhein C., Moras D., Romier C., Bolognesi M. (2013). Sequence-specific transcription factor NF-Y displays histone-like DNA binding and H2B-like ubiquitination. Cell.

[b0500] Noguero M., Atif R.M., Ochatt S., Thompson R.D. (2013). The role of the DNA-binding One Zinc Finger (DOF) transcription factor family in plants. Plant Sci..

[b0505] Nover L., Bharti K., Döring P., Mishra S.K., Ganguli A., Scharf K.-D. (2001). Arabidopsis and the heat stress transcription factor world: how many heat stress transcription factors do we need?. Cell Stress Chaperones.

[b0510] Nuruzzaman M., Sharoni A.M., Kikuchi S. (2013). Roles of NAC transcription factors in the regulation of biotic and abiotic stress responses in plants. Front. Microbiol..

[b0515] Oladosu Y., Rafii M.Y., Arolu F., Chukwu S.C., Muhammad I., Kareem I., Salisu M.A., Arolu I.W. (2020). Submergence Tolerance in Rice: Review of Mechanism, Breeding and, Future Prospects. Sustainability.

[b0520] Opdenakker K., Remans T., Keunen E., Vangronsveld J., Cuypers A. (2012). Exposure of Arabidopsis thaliana to Cd or Cu excess leads to oxidative stress mediated alterations in MAPKinase transcript levels. Environ. Exp. Bot..

[b0525] Opdenakker K., Remans T., Vangronsveld J., Cuypers A. (2012). Mitogen-activated protein (MAP) kinases in plant metal stress: regulation and responses in comparison to other biotic and abiotic stresses. Int. J. Mol. Sci..

[b0530] Pandey P., Irulappan V., Bagavathiannan M.V., Senthil-Kumar M. (2017). Impact of combined abiotic and biotic stresses on plant growth and avenues for crop improvement by exploiting physio-morphological traits. Front. Plant Sci..

[b0535] Petroni K., Kumimoto R.W., Gnesutta N., Calvenzani V., Fornari M., Tonelli C., Holt B.F., Mantovani R. (2012). The promiscuous life of plant NUCLEAR FACTOR Y transcription factors. Plant Cell.

[b0540] Qiao X., Li M., Li L., Yin H., Wu J., Zhang S. (2015). Genome-wide identification and comparative analysis of the heat shock transcription factor family in Chinese white pear (Pyrus bretschneideri) and five other Rosaceae species. BMC Plant Biol..

[b0545] Rabara R.C., Tripathi P., Rushton P.J. (2014). The potential of transcription factor-based genetic engineering in improving crop tolerance to drought. OMICS.

[b0550] Rajavashisth T.B., Taylor A.K., Andalibi A., Svenson K.L., Lusis A.J. (1989). Identification of a zinc finger protein that binds to the sterol regulatory element. Science.

[b0555] Rangan P., Furtado A., Henry R. (2020). Transcriptome profiling of wheat genotypes under heat stress during grain-filling. J. Cereal Sci..

[b0560] Rashad Y.M., Moussa T.A. (2020). Biocontrol Agents for Fungal Plant Diseases Management. *Cottage Industry of Biocontrol Agents and Their Applications*.

[b0565] Rushton D.L., Tripathi P., Rabara R.C., Lin J., Ringler P., Boken A.K., Langum T.J., Smidt L., Boomsma D.D., Emme N.J. (2012). WRKY transcription factors: key components in abscisic acid signalling. Plant Biotechnol. J..

[b0570] Rushton P.J., Somssich I.E., Ringler P., Shen Q.J. (2010). WRKY transcription factors. Trends Plant Sci..

[b0575] Ryan R.P., Vorhölter F.-J., Potnis N., Jones J.B., Van Sluys M.-A., Bogdanove A.J., Dow J.M. (2011). Pathogenomics of Xanthomonas: understanding bacterium–plant interactions. Nat. Rev. Microbiol..

[b0580] Santamaria M.E., Arnaiz A., Gonzalez-Melendi P., Martinez M., Diaz I. (2018). Plant perception and short-term responses to phytophagous insects and mites. Int. J. Mol. Sci..

[b0585] Santamaria M.E., Martínez M., Cambra I., Grbic V., Diaz I. (2013). Understanding plant defence responses against herbivore attacks: an essential first step towards the development of sustainable resistance against pests. Transgenic Res..

[b0590] Sato K., Kadota Y., Shirasu K. (2019). Plant immune responses to plant parasitic nematodes. Front. Plant Sci..

[b0595] Shahbaz M., Ashraf M. (2008). Does exogenous application of 24-epibrassinolide ameliorate salt induced growth inhibition in wheat (Triticum aestivum L.)?. Plant Growth Regul..

[b0600] Shahzad B., Fahad S., Tanveer M., Saud S., Khan I.A. (2019). Plant responses and tolerance to salt stress. *Approaches for enhancing abiotic stress tolerance in plants*.

[b0605] Shang X., Yu Y., Zhu L., Liu H., Chai Q., Guo W. (2020). A cotton NAC transcription factor GhirNAC2 plays positive roles in drought tolerance via regulating ABA biosynthesis. Plant Sci..

[b0610] Sharma P., Jha A.B., Dubey R.S., Pessarakli M. (2012). Reactive oxygen species, oxidative damage, and antioxidative defense mechanism in plants under stressful conditions. J. Bot..

[b0615] Sharma S., Kooner R., Arora R. (2017). Insect pests and crop losses. Breeding insect resistant crops for sustainable agriculture.

[b0620] Sharma V., Goel P., Kumar S., Singh A.K. (2019). An apple transcription factor, MdDREB76, confers salt and drought tolerance in transgenic tobacco by activating the expression of stress-responsive genes. Plant Cell Rep..

[b0625] Sharoni A.M., Nuruzzaman M., Satoh K., Shimizu T., Kondoh H., Sasaya T., Choi I.-R., Omura T., Kikuchi S. (2011). Gene structures, classification and expression models of the AP2/EREBP transcription factor family in rice. Plant Cell Physiol..

[b0630] Shen X.-J., Wang Y.-Y., Zhang Y.-X., Guo W., Jiao Y.-Q., Zhou X.-A. (2018). Overexpression of the wild soybean R2R3-MYB transcription factor GsMYB15 enhances resistance to salt stress and Helicoverpa armigera in transgenic Arabidopsis. Int. J. Mol. Sci..

[b0635] Singh S., Parihar P., Singh R., Singh V.P., Prasad S.M. (2016). Heavy metal tolerance in plants: role of transcriptomics, proteomics, metabolomics, and ionomics. Front. Plant Sci..

[b0640] Su L.-T., Li J.-W., Liu D.-Q., Zhai Y., Zhang H.-J., Li X.-W., Zhang Q.-L., Wang Y., Wang Q.-Y. (2014). A novel MYB transcription factor, GmMYBJ1, from soybean confers drought and cold tolerance in Arabidopsis thaliana. Gene.

[b0645] Sundgren T.K., Uhlen A.K., Lillemo M., Briese C., Wojciechowski T. (2018). Rapid seedling establishment and a narrow root stele promotes waterlogging tolerance in spring wheat. J. Plant Physiol..

[b0650] Tolosa L.N., Zhang Z. (2020). The Role of Major Transcription Factors in Solanaceous Food Crops under Different Stress Conditions: Current and Future Perspectives. Plants.

[b0655] Tripathi P., Rabara R.C., Rushton P.J. (2014). A systems biology perspective on the role of WRKY transcription factors in drought responses in plants. Planta.

[b0660] Ülker B., Somssich I.E. (2004). WRKY transcription factors: from DNA binding towards biological function. Curr. Opin. Plant Biol..

[b0665] Van Eck L., Davidson R.M., Wu S., Zhao B.Y., Botha A.-M., Leach J.E., Lapitan N.L. (2014). The transcriptional network of WRKY53 in cereals links oxidative responses to biotic and abiotic stress inputs. Funct. Integr. Genomics.

[b0670] Van Verk M.C., Pappaioannou D., Neeleman L., Bol J.F., Linthorst H.J. (2008). A novel WRKY transcription factor is required for induction of PR-1a gene expression by salicylic acid and bacterial elicitors. Plant Physiol..

[b0675] Wang C.-T., Ru J.-N., Liu Y.-W., Yang J.-F., Li M., Xu Z.-S., Fu J.-D. (2018). The maize WRKY transcription factor ZmWRKY40 confers drought resistance in transgenic Arabidopsis. Int. J. Mol. Sci..

[bib836] Wang X., Wang G., Guo T., Xing Y., Mo F., Wang H., Fan J., Zhang F. (2021). Effects of plastic mulch and nitrogen fertilizer on the soil microbial community, enzymatic activity and yield performance in a dryland maize cropping system. European J. Soil Sci..

[b0680] Wang Y., Zhang Y., Zhou R., Dossa K., Yu J., Li D., Liu A., Mmadi M.A., Zhang X., You J. (2018). Identification and characterization of the bZIP transcription factor family and its expression in response to abiotic stresses in sesame. PLoS ONE.

[b0685] Warmerdam S., Sterken M.G., Van Schaik C., Oortwijn M.E., Sukarta O.C., Lozano-Torres J.L., Dicke M., Helder J., Kammenga J.E., Goverse A. (2018). Genome-wide association mapping of the architecture of susceptibility to the root-knot nematode Meloidogyne incognita in Arabidopsis thaliana. New Phytol..

[b0690] Wassie M., Zhang W., Zhang Q., Ji K., Cao L., Chen L. (2020). Exogenous salicylic acid ameliorates heat stress-induced damages and improves growth and photosynthetic efficiency in alfalfa (Medicago sativa L.). Ecotoxicol. Environ. Saf..

[b0695] Wei X., Lu W., Mao L., Han X., Wei X., Zhao X., Xia M., Xu C. (2020). ABF2 and MYB transcription factors regulate feruloyl transferase FHT involved in ABA-mediated wound suberization of kiwifruit. J. Exp. Bot..

[b0700] Wu J., Lawit S.J., Weers B., Sun J., Mongar N., Van Hemert J., Melo R., Meng X., Rupe M., Clapp J. (2019). Overexpression of zmm28 increases maize grain yield in the field. Proc. Natl. Acad. Sci..

[b0705] Ximénez-Embún M.G., Castañera P., Ortego F. (2017). Drought stress in tomato increases the performance of adapted and non-adapted strains of Tetranychus urticae. J. Insect Physiol..

[b0710] Yanagisawa S. (2002). The Dof family of plant transcription factors. Trends Plant Sci..

[b0715] Yang J., Zhu J., Yang Y. (2017). Genome-wide identification and expression analysis of NF-Y transcription factor families in watermelon (Citrullus lanatus). J. Plant Growth Regul..

[b0720] Yang J.H., Lee K.H., Du Q., Yang S., Yuan B., Qi L., Wang H. (2020). A membrane-associated NAC domain transcription factor XVP interacts with TDIF co-receptor and regulates vascular meristem activity. New Phytol..

[b0725] Yang M., Chao J., Wang D., Hu J., Wu H., Gong D., Liu G. (2016). Genome-wide identification and expression profiling of the C2H2-type zinc finger protein transcription factor family in tobacco.. Hereditas.

[b0730] Yang O., Popova O.V., Süthoff U., Lüking I., Dietz K.-J., Golldack D. (2009). The Arabidopsis basic leucine zipper transcription factor AtbZIP24 regulates complex transcriptional networks involved in abiotic stress resistance. Gene.

[b0735] Yang Y., Zhou Y., Chi Y., Fan B., Chen Z. (2017). Characterization of soybean WRKY gene family and identification of soybean WRKY genes that promote resistance to soybean cyst nematode. Sci. Rep..

[b0740] Yang Z., Sun J., Chen Y., Zhu P., Zhang L., Wu S., Ma D., Cao Q., Li Z., Xu T. (2019). Genome-wide identification, structural and gene expression analysis of the bZIP transcription factor family in sweet potato wild relative Ipomoea trifida. BMC Genet..

[b0745] Yoshida T., Fujita Y., Sayama H., Kidokoro S., Maruyama K., Mizoi J., Shinozaki K., Yamaguchi-Shinozaki K. (2010). AREB1, AREB2, and ABF3 are master transcription factors that cooperatively regulate ABRE-dependent ABA signaling involved in drought stress tolerance and require ABA for full activation. Plant J..

[b0750] Yoshida T., Sakuma Y., Todaka D., Maruyama K., Qin F., Mizoi J., Kidokoro S., Fujita Y., Shinozaki K., Yamaguchi-Shinozaki K. (2008). Functional analysis of an Arabidopsis heat-shock transcription factor HsfA3 in the transcriptional cascade downstream of the DREB2A stress-regulatory system. Biochem. Biophys. Res. Commun..

[b0755] Yruela I. (2015). Plant development regulation: Overview and perspectives. J. Plant Physiol..

[b0760] Yruela I., Oldfield C.J., Niklas K.J., Dunker A.K. (2017). Evidence for a strong correlation between transcription factor protein disorder and organismic complexity. Genome Bio. Evo..

[b0765] Yu F., Liang K., Fang T., Zhao H., Han X., Cai M., Qiu F. (2019). A group VII ethylene response factor gene, ZmEREB180, coordinates waterlogging tolerance in maize seedlings. Plant Biotechnol. J..

[b0770] Yu J., Chai C., Ai G., Jia Y., Liu W., Zhang X., Bai T., Dou D. (2020). A Nicotiana benthamiana AP2/ERF transcription factor confers resistance to Phytophthora parasitica. Phytopathol. Res..

[b0775] Yuan X., Wang H., Cai J., Bi Y., Li D., Song F. (2019). Rice NAC transcription factor ONAC066 functions as a positive regulator of drought and oxidative stress response. BMC Plant Biol..

[b0780] Yuan X., Wang H., Cai J., Li D., Song F. (2019). NAC transcription factors in plant immunity. Phytopathol. Res..

[b0785] Yubing H., Min Z., Lihao W., Junhua W., Qiaoyan W., Rongchen W., Yunde Z. (2019). Improvements of TKC technology accelerate isolation of transgene-free CRISPR/Cas9-edited rice plants. Rice Sci..

[b0790] Yura T., Nakahigashi K. (1999). Regulation of the heat-shock response. Curr. Opin. Microbiol..

[b0795] Zeid I., Shedeed Z. (2006). Response of alfalfa to putrescine treatment under drought stress. Biol. Plant..

[b0800] Zhang D., Guo X., Xu Y., Li H., Ma L., Yao X., Weng Y., Guo Y., Liu C.M., Chong K. (2019). OsCIPK7 point-mutation leads to conformation and kinase-activity change for sensing cold response. J. Integr. Plant Biol..

[b0805] Zhao H., Wu D., Kong F., Lin K., Zhang H., Li G. (2017). The Arabidopsis thaliana nuclear factor Y transcription factors. Front. Plant Sci..

[b0810] Zhao N., Li C., Yan Y., Cao W., Song A., Wang H., Chen S., Jiang J., Chen F. (2018). Comparative transcriptome analysis of waterlogging-sensitive and waterlogging-tolerant Chrysanthemum morifolium cultivars under waterlogging stress and reoxygenation conditions. Int. J. Mol. Sci..

[b0815] Zheng X., Xing J., Zhang K., Pang X., Zhao Y., Wang G., Zang J., Huang R., Dong J. (2019). Ethylene response factor ERF11 activates BT4 transcription to regulate immunity to Pseudomonas syringae. Plant Physiol..

[b0820] Zhong R., Ye Z.-H. (2015). Secondary cell walls: biosynthesis, patterned deposition and transcriptional regulation. Plant Cell Physiol..

[b0825] Zhou Q.Y., Tian A.G., Zou H.F., Xie Z.M., Lei G., Huang J., Wang C.M., Wang H.W., Zhang J.S., Chen S.Y. (2008). Soybean WRKY-type transcription factor genes, GmWRKY13, GmWRKY21, and GmWRKY54, confer differential tolerance to abiotic stresses in transgenic Arabidopsis plants. Plant Biotechnol. J..

[b0830] Zhou W., Chen F., Meng Y., Chandrasekaran U., Luo X., Yang W., Shu K. (2020). Plant waterlogging/flooding stress responses: From seed germination to maturation. Plant Physiol. Biochem..

[b0835] Zhu Y., Evans K., Peace C. (2011). Utility testing of an apple skin color MdMYB1 marker in two progenies. Mol. Breed..

